# Breaking barriers: enhancing CAR-armored T cell therapy for solid tumors through microenvironment remodeling

**DOI:** 10.3389/fimmu.2025.1638186

**Published:** 2025-09-03

**Authors:** Tereza Andreou, Constantina Neophytou, Maria Kalli, Fotios Mpekris, Triantafyllos Stylianopoulos

**Affiliations:** ^1^ Cancer Biophysics Laboratory, Department of Mechanical and Manufacturing Engineering, University of Cyprus, Nicosia, Cyprus; ^2^ Cancer Genetics, Therapeutics & Ultrastructural Pathology Department, The Cyprus Institute of Neurology and Genetics, Nicosia, Cyprus

**Keywords:** CAR T cell, cell therapy, solid tumors, tumor microenvironment, extracellular matrix normalization

## Abstract

Whilst chimeric antigen receptor (CAR) T cell therapy has emerged as a revolutionary immunotherapeutic approach for hematological malignancies in recent years, several challenges remain to potentiate the efficacy of CAR T cell therapies for solid tumors. Here, we focus on the obstacles posed by the tumor microenvironment that hinder the effective trafficking, infiltration and precise tumor targeting by engineered cells. We discuss how the tumor microenvironment presents a physical barrier that needs to be surpassed for effective cell therapies and ongoing efforts in designing innovative CAR T cell therapies with enhanced tumor-targeting precision, improved stability, and overcoming on-target off-tumor toxicity are presented. We focus on recent advances in clinical and preclinical settings to reprogram the immunosuppressive tumor microenvironment, including stroma and blood vessel normalization strategies that can be leveraged to improve the tumor-homing and tumor-targeting potential of engineered therapeutic cells for immuno-oncology applications. As the endeavors for innovative CAR designs continue, we are entering an exciting era in the field of personalized cell therapies offering renewed hope to patients with hard-to-treat solid tumors.

## Introduction

The immune system is a double-edged sword in cancer development and progression but can also be harnessed for cancer therapy. One major advancement is the adoptive transfer of T cells engineered with chimeric antigen receptors (CARs), which has shown significant success in treating various tumors, especially in hematological malignancies, with seven CAR T cell products approved in the US currently (1). CAR T cells are genetically engineered T lymphocytes designed to recognize and eliminate tumor cells through synthetic receptors that combine an antibody-derived single-chain variable fragment (scFv) for antigen specificity with intracellular signaling domains that mediate T cell activation and effector functions (2, 3). Contrary to hematological cancers, solid tumors are highly heterogeneous and comprise an intricate tumor microenvironment (TME), which presents formidable obstacles to the efficacy of CAR T cell therapies (4). The TME is a highly structured ecosystem containing cancer cells surrounded by tumor stroma tissue, encompassing a diverse population of cancer-associated cells (fibroblasts, mesenchymal cells, immune cells, endothelial cells, pericytes), an abnormal tumor vasculature, and a dense extracellular matrix (ECM). Collectively, this complex and dynamic ecosystem presents a challenging barrier that greatly limits the efficacy of CAR T cell therapies in solid tumors. To develop effective CAR T cell therapies, the engineered cells need to be able to (i) successfully infiltrate the TME through the dense ECM, (ii) survive the hostile environment (hypoxia, reduced nutrient availability, immunosuppressive factors, intratumoral mechanical stresses), and (iii) effectively and selectively target cancer cells (5, 6). In this review article, we discuss recent advancements in CAR T cell engineering to overcome these obstacles, focusing on key aspects of tumor patho-physiology and innovative design strategies, as well as combination treatments to tackle tumor heterogeneity using strategies that target multiple tumor-specific antigens/tumor-associated antigens (TSAs/TAAs).

## CAR T cell therapies in the clinic

The emergence of CAR T cell therapy has marked a significant turning point in the field of oncology, especially in the treatment of hematologic malignancies such as leukemia, lymphoma, and multiple myeloma (1). A crucial aspect of bringing CAR T cell therapy into clinical practice has been the extensive research conducted through clinical trials, which serve as the primary mechanism for testing the feasibility, optimizing the design, and ensuring safety of CAR-engineered cells in human subjects. These trials have provided essential insights into how CAR-engineered cells behave in the human body, how effectively they eliminate cancer cells, and the adverse effects they might cause. They have also allowed for the exploration of different CAR constructs, cell sources (autologous versus allogeneic), target antigens, combination strategies and, more recently, different host cells, all of which contribute to refining the therapy for broader application. Over the past decade, the clinical trial landscape for CAR T cell therapy has expanded rapidly, with numerous studies progressing from early-phase safety evaluations to large-scale efficacy trials (**Table 1**) that directly influence regulatory approvals and clinical guidelines.

**Table 1:** Completed Clinical Trials using CAR T cells and their outcomes.

ClinicalTrials.gov identifier	Target Antigen	Cancer Type	Phase	Key outcomes	Reference
NCT02435849	CD19	B-cell Acute Lymphoblastic Leukemia	II	81% complete remission rate in pediatric/young adult patients	(7)
NCT02348216	CD19	Diffuse Large B-cell Lymphoma	II	51% complete remission rate, manageable safety profile	(8)
NCT03056339	CD19	Lymphoid Malignancies	I/II	73% overall response rate; no cytokine release syndrome or neurotoxicity; promising for off-the-shelf applications	(9)
ChiCTR2000040645	CD19 and GCC	Metastatic Colorectal Cancer	I	40% objective response rate; Median overall survival 22.8 months	(10)
NCT03233854	CD19 and CD22	Large B-cell Lymphoma	I	62% of patients responded with 29% complete remission; No dose-limiting toxicities	(11)
NCT01869166	EGFR	Metastatic Pancreatic Cancer	I	28% patients with partial response, 58% with stable disease and 14% with disease progression; Grade ≥3 adverse events	(12)
NCT02541370	CD133	Hepatocellular Carcinoma/Colorectal Cancer/Pancreatic Cancer	I	Among 23 patients, 3 achieved partial remission and 14 had stable disease, resulting in a 3-month disease control rate of 65.2% and a median progression-free survival of 5 months.	(13)
NCT03361748	BCMA	Relapsed/Refractory Multiple Myeloma	II	73% overall response rate; 33% stringent complete remission rate	(14)
NCT03548207	BCMA	Relapsed/Refractory Multiple Myeloma	Ib/II	97% overall response rate; deep, durable responses in heavily pretreated patients	(15)
NCT04404595	CLDN18.2	Advanced Gastric and Pancreatic Adenocarcinoma	Ib	1 patient complete response, 2 partial response, 2 stable disease 3 progression of disease; No dose limiting toxicities; No Grade 4 adverse effects	(16)
NCT00902044	HER2	Sarcoma/Glioblastoma	I	Some stable disease; limited persistence; safety challenges	(9)
NCT01935843	HER2	Biliary Tract Cancer/Pancreatic Cancer	I	18% evaluable patients with stable disease; safe without significant adverse events	(17)
NCT00085930	GD2	Relapsed/Refractory Neuroblastoma	I	Modest efficacy (18.2%) in those with active disease at infusion; More promising durability (62.5%) in those treated while in remission but at high risk.	(18)
NCT02159716	MSLN	Malignant Pleural Mesothelioma/Ovarian carcinoma/Pancreatic Cancer	I	60% patients with progressive disease; 40% patients with stable disease; no evidence for on-target toxicities; modest expansion with limited persistence in the blood	(19)

The first significant clinical breakthroughs in CAR T cell therapy emerged from studies focused on CD19, a protein commonly found on the surface of B cells and widely expressed in various B-cell malignancies. Among the pioneering efforts, a landmark clinical trial carried out by researchers at the University of Pennsylvania, in partnership with Novartis, played a pivotal role in bringing the first-ever CAR T cell therapy to the market. This collaborative research ultimately resulted in the creation and clinical advancement of Tisagenlecleucel (commercially known as Kymriah) - the first CAR T cell therapy to gain regulatory approval for use in human (7). In this Phase II trial (NCT02435849), 81% of children and young adults with relapsed or refractory B-cell acute lymphoblastic leukemia (B-ALL) achieved complete remission after receiving CAR T cell therapy. These promising outcomes led to the first FDA approval of a CAR T cell therapy in 2017. Similarly, Axicabtagene ciloleucel (Yescarta) demonstrated a 51% complete response rate in adults with relapsed/refractory diffuse large B-cell lymphoma (DLBCL) in the ZUMA-1 trial (NCT02348216), supporting its approval shortly thereafter (8).

Following the initial success of CAR T cell therapies directed against the CD19 antigen, the scope of clinical investigations has broadened to include a variety of alternative tumor-associated antigens. This expansion has been especially prominent in the field of multiple myeloma, a cancer of plasma cells, where researchers have identified B-cell maturation antigen (BCMA) as a highly promising therapeutic target due to its consistent expression on malignant plasma cells and limited presence on normal tissues (20, 21). One of the most notable advances in this area has come from the KarMMa clinical trial (NCT03361748), which evaluated the efficacy of a BCMA-targeted CAR T cell product known as Idecabtagene vicleucel (marketed as Abecma) (14). The results of this pivotal study were highly encouraging: the therapy produced an overall response rate (ORR) of 73%, meaning nearly three-quarters of participants with relapsed or refractory disease experienced a measurable reduction in tumor burden. Moreover, 33% of those patients achieved a stringent complete response, indicating the complete disappearance of detectable cancer, a particularly impressive outcome given the advanced disease status of the enrolled population. Another BCMA-targeted CAR T therapy, Ciltacabtagene autoleucel (marketed as Carvykti), showed an impressive 97% overall response rate in the CARTITUDE-1 trial (NCT03548207), with durable responses observed in a heavily pretreated population (15).

Although the most notable progress with CAR T cell therapy has been achieved in the treatment of hematologic malignancies, researchers have increasingly turned their attention towards exploring the application of CAR-engineered cells in solid tumors, which present a more complex therapeutic challenge. A growing number of early-phase clinical trials are underway to assess the safety and efficacy of CAR T cells engineered to recognize tumor-associated antigens specific to solid cancers. Among these, targets such as human epidermal growth factor receptor 2 (HER2) in certain types of sarcomas, breast, gastric and ovarian cancers (NCT00902044, NCT04995003 and NCT04511871), cleaved Mucin1 in metastatic breast cancer (NCT04020575), mesothelin in pancreatic and ovarian cancers, and disialoganglioside (GD2) in neuroblastoma (NCT03373097) have attracted considerable interest (22). Despite these efforts, clinical outcomes in solid tumors have so far been relatively modest compared to the dramatic responses observed in hematological cancers. For example, a Phase I trial (NCT00902044) involving HER2-targeted CAR T cells administered to patients with sarcoma demonstrated that while a subset of patients achieved disease stabilization, the therapy faced significant hurdles (9). The challenges in solid tumors include heterogeneous antigen expression, the immunosuppressive TME, mechano-transduction pathways, and inefficient T cell trafficking, all of which hinder therapeutic efficacy (23). To overcome these barriers, more recent clinical trials are exploring “armored” CAR T cells, for instance engineered to secrete cytokines like IL-12, or dual-targeting CARs designed to prevent antigen escape (e.g. NCT06343376). Other studies are testing CAR natural killer (CAR NK) cells (e.g. NCT03056339) and CAR macrophages (CAR Ms; NCT04660929), which may offer advantages such as reduced toxicity and off-the-shelf production potential (24). Trials involving allogeneic CAR T cells, such as those developed by Allogene Therapeutics and Cellectis (e.g. NCT04696731, NCT01430390 and NCT04416984), are under investigation to address scalability and manufacturing limitations of autologous CAR T therapies (25).

In addition to evaluating therapeutic efficacy, ensuring patient safety remains a critical priority in the clinical development of CAR T cell therapies. Among the most commonly observed and potentially serious side effects are cytokine release syndrome (CRS) and immune effector cell-associated neurotoxicity syndrome (ICANS) - two immune-mediated toxicities that can range in severity from mild to life-threatening (26). CRS results from the rapid activation and proliferation of CAR-modified immune cells, leading to a surge of inflammatory cytokines, while ICANS involves neurological complications such as confusion, seizures, or cerebral edema, believed to be linked to immune cell activity in the central nervous system. Recognizing the prevalence and impact of these adverse events, clinical trials have increasingly adopted proactive safety protocols to manage and mitigate risks. These typically include the administration of tocilizumab, an IL-6 receptor antagonist that helps counteract the inflammatory cascade associated with CRS, as well as the use of corticosteroids to suppress overactive immune responses when necessary (27). Furthermore, researchers have been enhancing the safety profile of CAR T cell therapies by engineering built-in regulatory mechanisms, such as “suicide switches”, which allow clinicians to selectively deactivate or eliminate the CAR-modified cells in cases of severe toxicity (28, 29).

Clinical trials continue to evolve, with Phase III trials underway to compare CAR T cell therapies against standard of care treatments in second-line settings (30). Additionally, combinations with immune checkpoint inhibitors (ICIs), oncolytic viruses, or kinase inhibitors are being tested to further enhance the efficacy of CAR-engineered cells (31–34). These ongoing studies not only aim to broaden the scope of CAR T cell therapies but also refine safety and accessibility aspects, setting the stage for the next generation of cell therapies for solid tumors.

## Towards the design of innovative CAR-engineered cells to target solid tumors

The preclinical studies that guided the development of the above approved therapies for hematological cancers revealed important insights into how CAR T cell designs influence clinical performance. For example, axicabtagene ciloleucel, using a CD28 domain, was optimized for rapid cytokine production and early expansion (35), whereas tisagenlecleucel which incorporates a 4-1BB domain, was selected for its ability to support long-term persistence and memory formation (36). These features correlate with the clinical profiles of the currently approved products. Axicabtagene ciloleucel demonstrates faster proliferation but higher toxicity risk (35), while tisagenlecleucel tends to show lower toxicity with prolonged activity. Lisocabtagene maraleucel (marketed as Breyanzi) emphasized the synergy between CD4^+^ and central memory CD8^+^ T cells to improve durability (37), and idecabtagene vicleucel’s BB2121 construct was selected for strong *in vivo* cytotoxicity and tumor clearance (14, 38). These findings underscore the predictive value of robust preclinical assays focused on long-term antitumor activity, memory phenotype, and cytokine production.

While many clinical trials are ongoing to evaluate the efficacy or CAR T cells in solid tumors (e.g., lung, breast, pancreatic, and glioblastoma), none have yet received regulatory approval to date (39). The application of CAR-engineered cells remains constrained by tumor heterogeneity, limited tumor infiltration, and immunosuppressive microenvironments (40). To enhance the therapeutic effectiveness of CAR T cell therapies, numerous innovative CAR designs have been created to boost antitumor activity and address treatment resistance caused by insufficient expansion, infiltration, and persistence of CAR-engineered cells (41–44). Over time, five generations of CARs have been developed, each aimed at addressing the limitations of earlier constructs and enhancing therapeutic efficacy.

First-generation CARs included a basic structure composed of an extracellular antigen-binding domain linked to a CD3ζ signaling domain. However, they demonstrated limited clinical activity due to insufficient T cell activation (45). To overcome this, second- and third-generation CARs introduced one or two costimulatory domains—most commonly CD28, CD137 (4-1BB), or both in the case of third-generation constructs resulting in enhanced T-cell activation, memory formation, and persistence. Among these, 4-1BB has shown particular advantage in sustaining long-term CAR T cell functionality *in vivo*, contributing to prolonged tumor control (46). Yet, these designs showed limited success in solid tumors.

To overcome these limitations, the fourth and fifth (or next)-generation of CARs was developed. The fourth generation of CARs builds upon the second-generation by incorporating a protein, such as interleukin 12 (IL-12), which is expressed either constitutively or upon CAR activation. T cells engineered with these fourth-generation CARs are called T cells redirected for universal cytokine-mediated killing (TRUCKs) (47). Activation of these CARs triggers the production and release of the targeted cytokine, enhancing tumor destruction through various synergistic mechanisms, including exocytosis (perforin, granzyme) or death ligand–death receptor (Fas–FasL, TRAIL) systems, helping to remodel the TME and recruit additional immune responses, especially useful in the context of solid tumors (48). Fifth-generation CAR T cells build on the second-generation design and include a truncated cytoplasmic IL-2 receptor β-chain domain with a binding site for the transcription factor STAT3. The antigen-specific activation of this receptor simultaneously stimulates the T cell receptor (TCR; via the CD3ζ domains), co-stimulatory (CD28 domain), and cytokine (JAK–STAT3/5) signaling, which provides all three necessary synergistic signals to drive complete T cell activation and proliferation. This latest generation aims to enhance persistence and overcome exhaustion, particularly in hostile tumor microenvironments (49). Together, these advances reflect a progressive refinement of CAR-engineered cell function, safety, and applicability across cancer types.

In parallel with generational improvements, numerous complementary strategies have been developed to enhance CAR T cell therapies, though they are not defining features of later-generation constructs. These include dual or pooled CAR-engineered cells to address antigen heterogeneity (50), artificial intelligence- guided tools such as radiomics for improved target selection (51, 52), and modifications to enhance tumor infiltration such as chemokine receptor expression (53), local administration techniques (e.g. intratumoral injection (54), microneedle patches (55), and matrix-degrading enzyme secretion (56). Moreover, combining CAR-engineered cells with chemotherapy or externally administered ICIs, such as anti-PD-1 or anti-CTLA-4 antibodies, offers promising synergy but represents therapeutic strategies adjunct to CAR design (57–59). While these innovations contribute significantly to efficacy and applicability, they are best viewed as supportive enhancements rather than intrinsic features of fourth- or fifth-generation CAR T cell platforms.

The evolution of CAR designs has been instrumental to the success of the currently approved CAR T cell therapies for hematological cancers. Moving forward, combining innovative CAR designs with strategies to break down the physical barriers in solid tumors will lead to the next-generation of CAR cell therapies. In the following sections, we shift our focus on the patho-physiological barriers and strategies to overcome these barriers and enhance the therapeutic efficacy of CAR-engineered cells in solid tumors.

## Patho-physiological abnormalities in solid tumors: Drivers of progression and barriers to therapy

**Figure 1:**
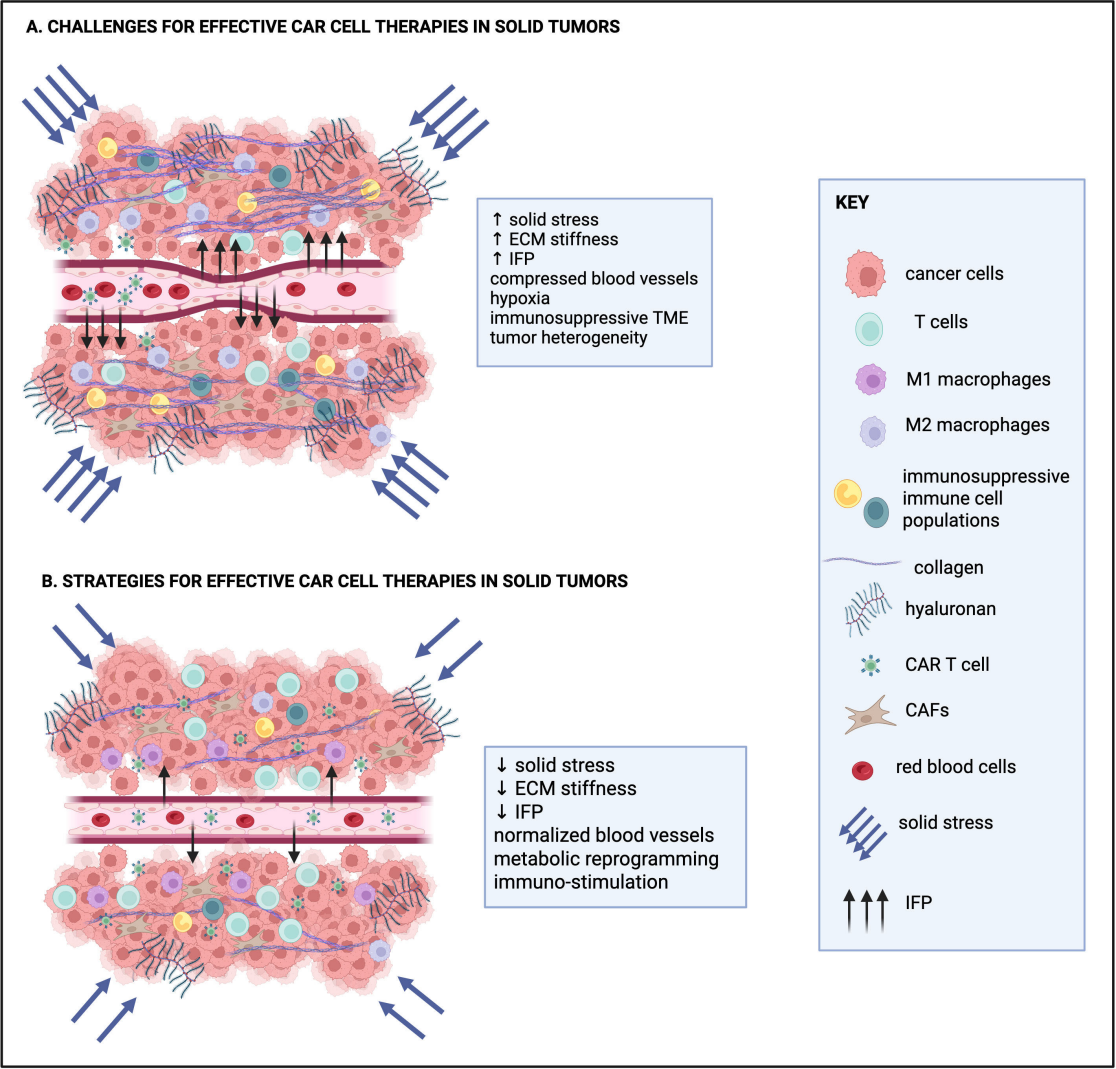
Current challenges for effective CAR T cell therapies in solid tumors and strategies to overcome them. **(A)** Challenges of CAR T cell therapies for solid tumors include increased solid stress, the stiff extracellular matrix (ECM), high interstitial fluid pressure (IFP) and compressed blood vessels that collectively hinder the effective trafficking of CAR T cell therapies to solid tumors. The hypoxic and immunosuppressive tumor microenvironment (TME) and tumor heterogeneity also contribute to the limited therapeutic efficacy of CAR T cell therapies in solid tumors. **(B)** Current efforts for effective CAR cell therapies focus on strategies to reduce solid stress and normalize IFP, ECM density, ECM and blood vessel structure, as well as metabolic and immune cell reprogramming. Collectively, these approaches are aimed at increasing infiltration and persistence of CAR-engineered cells in solid tumors. Created in https://BioRender.com.

In contrast to hematological malignancies, where cancer cells circulate freely in the blood and lymphatic systems, tumor cells in solid tumors are embedded within a complex matrix composed of both cellular and non-cellular components (**Figure 1**). In many of these tumors, the TME is marked by a desmoplastic reaction that contributes to ECM stiffening, the only mechanical aspect of a tumor clinicians and patients can sense. Desmoplasia involves activation of cancer-associated fibroblasts (CAFs) producing large amounts of extracellular proteins that form a dense matrix around and within the tumor. High deposition of fibrillar proteins and macromolecules, such as type I collagen and hyaluronan, along with secretion of crosslinking enzymes (e.g. lysyl oxidases; LOXs) and CAFs’ contractile forces, remodel the matrix and increase its stiffness (60). As cancer cells proliferate rapidly within this stiff ECM, they expand at the expense of the surrounding host tissue, which in turn applies reciprocal compressive forces to resist tumor growth (61–63). This results in the accumulation of compressive mechanical stress, also known as *solid stress* (i.e., force per unit area), within the tumor (61, 64–66).

At the cellular level, elevated ECM stiffness and solid stress regulate the function of cancer cells and CAFs, including cell cycle progression, invasion, cytokine secretion, survival and hence therapy resistance (extensively reviewed in (67, 68)). At the tissue level, they compress blood and lymphatic vessels, restricting the transport of oxygen, nutrients, and therapeutic agents into the tumor, and thereby contributing to hypoxia (69–71). Hypoxia, in turn, promotes tumor progression and resistance to therapy. In response to the hypoxic environment, new blood vessels are formed to supply oxygen to the rapidly growing tumor. However, these vessels are often immature, characterized by reduced pericyte coverage, detachment or loss of the basement membrane, and large gaps between endothelial cells. As a result, they are leaky and allow excessive fluid to escape from the vasculature into the tumor extracellular space, leading to elevation of the *interstitial fluid pressure* (IFP), which can also impose shear stress on structural components of the TME (72). IFP elevation can further impair the intratumoral distribution of essential molecules, further limiting the effectiveness of a potential treatment, including immunotherapy-based approaches (70, 73).

However, despite growing recognition that mechanical cues in the TME not only impair drug delivery but also influence cancer cell behavior, their impact on immune cell function remains poorly understood. The regulation of tumor immunity relies on complex interactions among immune cells, stromal components such as CAFs and the vasculature; yet how mechanical forces shape these cellular interactions is still being elucidated. This knowledge gap is particularly critical in the context of immunotherapies, including CAR-based approaches, which may be compromised by both the altered immune dynamics and the physical barriers imposed by the tumor stroma. Moreover, immune cells rely on direct physical interactions to recognize and eliminate malignant cells, making them highly sensitive to mechanical features of their environment. While several studies have investigated how mechanical signals affect T cell behavior, most have focused on nanoscale level- or shear- forces (74). In contrast, the effects of tissue-level mechanical stresses, such as compression and elevated IFP, on tumor–immune interactions remain underexplored, despite their potential to significantly limit the efficacy of current and emerging immunotherapies.

## Mechanical regulation of immune dynamics: implications for CAR T cell therapies

Immune cells rely significantly on direct physical interactions both with cancer cells and with other components of the TME, to carry out their functions. From forming immune synapses to trafficking through tissues and initiating cytotoxic responses, these processes are inherently mechanical in nature. As such, any alteration in the physical properties of the TME can influence immune cell localization, activation, and effector function. In solid tumors, mechanical abnormalities such as elevated IFP, increased ECM stiffness, and compressive solid stresses are hallmark features. In the following sections, we examine how these physical stimuli regulate anti-tumor immunity by (i) directly regulating immune cell responses and (ii) indirectly regulating immune-stromal and immune-tumor interactions.

### Direct force-induced immune cell responses

*T cells* rely on physical interactions for antigen recognition and activation through the TCR. Mechanical forces can modulate TCR conformations and enhance its interaction with CD3, promoting cytotoxic responses (75, 76). Additionally, mechanical signals such as matrix stiffness can suppress T cell metabolism via YAP signaling, potentially impairing their activity in stiff tumor tissues (77). Regulatory T cells (Tregs), which suppress immune responses, are also affected by ECM components like hyaluronan, influencing their immunosuppressive capacity (78). In a similar manner*, B cells*, through their B cell receptors (BCRs), can sense and respond to mechanical forces. It has been shown that low-level mechanical cues facilitate BCR activation via lipid signaling, though its relevance in the TME remains unclear (79). *Macrophages* also demonstrate significant mechanosensitivity. Pro-inflammatory M1 macrophages can be activated by mechanical stretching via the FAK/NF-κB pathway (80), or by stiff ECM through YAP and Piezo1 pathways (81, 82). Vice versa, macrophages can stiffen the ECM by secreting collagen crosslinkers creating a positive feedback loop (83). IFP can polarize macrophages toward either pro- or anti-tumor phenotypes, with flow-driven integrin/Src signaling enhancing their tumor-promoting behavior. Dendritic Cells (DCs), key antigen-presenting cells, are sensitive to fluid shear stress, which regulates their migration, metabolic activity, and expression of activation markers like MHC I (84, 85). These responses suggest that interstitial flow in tumors could influence DC function and thus, the initiation of anti-tumor immune responses (86, 87). Finally, Natural Killer (NK) cells also respond to mechanical cues. Shear stress enhances the secretion of NK-derived extracellular vesicles containing cytotoxic molecules like granzyme B and perforin, which mediate tumor cell killing (88). However, the effects of mechanical forces on NK cell activation remain underexplored.

### Indirect force-induced immune-tumor and immune-stroma interactions

The dense ECM and compressed vasculature act as physical barriers to immune cell infiltration, contributing to the development of an immune-excluded phenotype (70). The solid stress-induced hypoxia could also induce metabolic rewiring in tumor cells and CAFs toward glycolysis via HIF-1α-mediated reprograming eventually leading to rapid TME acidification, which could induce T cell inhibition via decreasing expression of major TCR components, reduced secretion of IL-2, tumor necrosis factor alpha (TNFα) and interferon-gamma (IFNγ) and upregulated expression of immune checkpoints (89–91). The elevated IFP promotes the outward flow of tumor- and stromal cell–derived immunosuppressive exosomes, which accumulate at the tumor boundary. These exosomes may attract immunosuppressive immune cells, further supporting tumor progression (92). The elevated IFP could also upregulate the expression of transforming growth factor-β (TGF-β) in tumor cells, which is a major driver of immune dysfunction through recruiting Treg cells and inhibiting CD8^+^ and helper T cells (93–95). Also, TGF-β attracts immune cells such as NK cells and macrophages to the tumor site but it impairs their anti-tumor functions (96). The IFP-regulated TGF-β signaling is also a contributing mechanism to the antigen heterogeneity in solid tumors, as it can downregulate epithelial cell adhesion cofactor (EpCAM) expression on the surface of tumor cells. EpCAM is a common tumor-associated antigen for the development of CAR T based cell therapies, and hence, the IFP-induced downregulation of this molecule would impair the recognition of engineered cells (97). Activated Tregs can also secrete TGF-β, further enhancing immune suppression (98). Mechanical cues in the TME, including interstitial flow and contractile forces exerted by CAFs, can also activate latent TGF-β, rendering immune cells inactive (99).

In the context of TME and under the influence of mechanical cues, cancer cells directly interact with diverse types of immune cells regulating their anti-tumor properties. For example, mechanical tension during the immune synapse between T cells and cancer cells, along with increased stiffness of repopulating tumor cells, enhances T cell–mediated killing by promoting perforin pore formation on the cancer cell membrane (100). Similarly, increased hydrostatic pressure applied to cancer cells has been shown to induce expression of immunogenic cell death markers, including calreticulin. When such mechanically stressed cancer cells were co-cultured with DCs, there was increased DC activity (phagocytosis), enhanced recruitment of tumor-specific T cells, and a reduction in Treg cell populations (101). However, not all mechanical cues are immunostimulatory and thus, they could interfere with the mechanism of action of several CAR based approaches. The implantation of pre-compressed breast cancer cells into mice led to immunosuppression, characterized by elevated M2 macrophages and reduced active T cells within the TME. This effect was mediated by PD-L1–containing exosomes released by cancer cells in response to compression (102). Autophagy, which is a stress-induced mechanism activated in cancer cells, facilitates degradation of intracellular components, including MHC-I and granzyme B, potentially impairing recognition and apoptosis by T and NK cells (103–105). Paradoxically, autophagy may also degrade PD-1/PD-L1 checkpoint molecules, which could enhance T cell–mediated killing (106).

Altogether, the patho-physiological properties of the TME present significant barriers both at the tissue and cellular level to the success of immunotherapies, including CAR-based immunotherapies which are currently administered in an environment that is resistant to immune-mediated clearance. While some mechanical stimuli, such as fluid flow, may enhance the activation of innate immune cells like macrophages or DCs, these effects are context-dependent and not always beneficial in the tumor setting. On the cancer cell side, mechanical forces trigger mechanisms that enhance tumor cell survival and resistance to immune attack. Overall, the mechanical landscape of solid tumors tends to foster immune evasion, contributing to the limited efficacy and persistence of CAR-based therapies in these contexts. Overcoming these patho-physiological barriers will require novel strategies, including ECM-modifying agents, co-targeting of immunosuppressive pathways (such as TGF-β blockade), the design of CAR constructs or delivery platforms optimized for function in mechanically stressful environments. A deeper understanding of how mechanical forces regulate immune cell behavior will be critical for advancing next-generation CAR cell therapies that are more effective in the complex environment of solid tumors.

## Current applications and innovative CAR designs to overcome patho-physiological challenges in solid tumors.

### Strategies to enhance CAR T cell infiltration through the dense ECM

As elucidated above, the dense tumor ECM poses a serious obstacle for applying engineered CAR T cell therapies in solid tumors. Both the dense/stiff and immunosuppressive nature of the tumor ECM can impede the engineered CAR T cells to enter the tumor, migrate towards the target cells, and subsequently kill the cancer cells. Moreover, recently published data show that the tumor ECM can lead to T cell exhaustion and therefore can significantly reduce the efficacy of CAR T cell therapies in solid tumors (107). A number of innovative approaches are currently being tested to enhance CAR T cell infiltration through the dense ECM, mainly focusing on ECM remodeling strategies, as discussed below (also **Figure 1**). These approaches fall into two main categories: i) strategies whereby CAR-engineered cells are pre-treated *ex vivo* to improve their infiltration through the dense ECM and, ii) treatment with agents that target ECM components with the aim to normalize ECM stiffness and improve immunostimulation.

In terms of the *ex vivo* pre-treatment of CAR-engineered cells, several strategies are currently being explored such as pre-conditioning and inhibitory treatments of T-lymphocytes, the use of artificial hydrogels and genetic engineering of CAR immune cells to enhance their infiltration and targeting in solid tumors. Hypoxic preconditioning of NK cells enhances their migration and cytotoxicity (108, 109); a similar approach may benefit CAR T cells by boosting stress-adaptive responses and ECM navigation. Interestingly, vinblastine pre-treatment of cytotoxic T-lymphocytes provides them with a better capability of migrating through dense 3D collagen matrix along with more effective killing of cancer cells in an *in vitro* model (110). The results by Zhao et al., suggest that pharmacological pre-treatment with vinblastine may be adopted for CAR T cells as a very simple *ex vivo* manipulation, to successfully target ECM-enriched solid tumors. Recently, Zhang et al., showed that ECM stiffness-associated biomechanical stress and the stress-responsive involvement of Osr2 in HDAC3-mediated alterations in the epigenetic regulation of CAR T cells promote their exhaustion within solid tumors (111). These results suggest that pre-treatment of CAR T cells with certain inhibitors targeting Osr2 or HDAC3 may partially delay the terminal CAR T cell exhaustion, thereby increasing the efficacy of CAR T cell therapy toward solid tumors. Furthermore, T cells preconditioned in hyaluronic acid (HA)-based hydrogels or ECM-mimicking hydrogels show enhanced expansion and functionality (112). HA hydrogels can also deliver CAR T cells directly into tumor cavities, potentially bypassing ECM barriers and preventing tumor recurrence, but more trials are required to confirm efficacy (113). CAR T cells have also been engineered to secrete ECM-degrading enzymes like heparanase, hyaluronidase, or collagenase to improve tumor infiltration and antitumor activity (114). Notably, the expression of heparanase is often downregulated in *ex vivo* expanded T cells due to epigenetic silencing, for example via p53-mediated suppression, but can be restored through genetic engineering, significantly enhancing their ECM invasion capacity and chemokine-mediated migration (115). In addition, CAR T cells have been designed to recognize and target ECM components such as the fibronectin EDA domain, chondroitin sulfate proteoglycan 4, or tenascin C, and such approaches have shown improved migration, infiltration, and tumor killing in preclinical models (116–118).

It is well established that T cells mechanically probe their environment, adjusting their response based on the stiffness of target cells *in vivo* and ligand-carrying surfaces *ex vivo*. Recently, Yassin et al., engineered a novel mechanostimulatory platform for T-cell activation based on an antigen-carrying surface with controlled elasticity and nanotopography (119). This platform is designed to optimize and balance T-cell exhaustion, proliferation, and CAR expression and it enhances the differentiation of T cells into the central memory subset, which is crucial for the persistence of CAR T cell therapy’s anticancer effects. Crucially, this platform produces CAR T cells with higher antitumor efficacy, as validated through *ex vivo* experiments, and with higher *in vivo* persistence and ability to suppress tumor proliferation, as compared to CAR T cells generated by standard protocols.

### ECM normalization strategies to overcome intratumoral solid stress

Several promising strategies have emerged to modulate the intratumoral solid stress and improve the therapeutic outcomes of CAR T cell treatment in solid tumors. One strategy relies on the selective degradation of ECM components like collagen, HA, and heparan sulfate proteoglycans using enzymes such as collagenase, hyaluronidase and heparinase, respectively. These enzymes help dismantle the dense ECM structure, reduce solid stress and promoting deeper T cell penetration (114). Although direct injections of bacterial collagenase were initially explored, their high immunogenicity limited further development (120). In contrast, nattokinase injections have successfully degraded fibronectin, reduced tumor stiffness, and enhanced CAR T cell infiltration in animal models (121). To avoid the need for injections, researchers have also designed nanodevices that release ECM-degrading enzymes like hyaluronidase in response to the acidic tumor environment or in response to thermoregulation, thereby facilitating immune cell infiltration (122). Furthermore, other pharmacological agents such as losartan and ketotifen have been shown to act as mechano-modulators of the TME, lowering IFP and enhancing perfusion, all of which are permissive to CAR T cell trafficking and infiltration (123–126). Moreover, innovative approaches such as engineering macrophages (CAR-147 macrophages) to secrete ECM-degrading enzymes or using IL-12-engineered T cells to eliminate CAFs have demonstrated the ability to remodel the TME in favor of T cell infiltration (127, 128). In addition, biotechnological and nanotechnological innovations are providing new tools to remodel the tumor ECM. Devices such as *in situ* gold bioreactors that alter ECM viscosity after microwave ablation and nanoengineered CAR T cell hybrids linked to photosensitizers offer ways to locally disrupt the ECM upon external triggers like laser irradiation (129, 130). These emerging technologies, along with further collaborations between genetic engineers and biotechnologists, are expected to expand the applicability and effectiveness of CAR T cell therapy against solid tumors in the future.

Targeting tumor-associated fibrosis, particularly that driven by CAFs, offers another avenue for modulating the TME (131). Fibrotic responses, largely mediated through the CXCL12/CXCR4 axis, result in excessive ECM deposition and physical exclusion of cytotoxic T lymphocytes (132). Pharmacological inhibition of the CXCL12/CXCR4 axis, such as through plerixafor, has demonstrated the potential to alleviate fibrosis and restore immune access to tumor regions (133). Inhibiting fibrosis-related pathways, such as through the use of focal adhesion kinase (FAK) inhibitors or blockers of hyaluronan synthesis, has been shown to soften the tumor stroma and enhance CAR T therapy (134). Similarly, targeting key fibrosis regulators like TGF-β, HSP47, extracellular HSP90, LOXs, and transglutaminases are being explored as a means to prevent the formation of dense ECM barriers and improve CAR T cell efficacy (135).

Lastly, due to the critical role of matrix stiffness in modulating immune responses, targeted reduction of ECM rigidity, via inhibition of matrix crosslinking enzymes such as lysyl oxidase-like 2 (LOXL2) or direct CAR T targeting of CAFs expressing fibroblast activation protein (FAP), has also emerged as an effective strategy (136–138). Anti-FAP CAR T cells have been shown to reduce stromal density, decrease vascular abnormalities, and promote endogenous T cell infiltration, resulting in substantial tumor regression in preclinical models (139). However, excessive degradation of ECM can paradoxically impair T cell migration by eliminating necessary physical cues, underscoring the need for balanced modulation. Overall, these combined biophysical and biochemical strategies to re-engineer the TME hold great promise for enhancing CAR T cell therapy efficacy against solid tumors, and merit further investigation through rigorous preclinical and clinical studies.

### Blood vessel normalization strategies

Normalizing tumor vasculature is another promising strategy to reduce IFP and improve immune cell delivery. Anti-angiogenic therapies, directly or indirectly targeting vascular endothelial growth factor (VEGF) or its receptor (VEGFR), including clinically approved agents such as bevacizumab and sunitinib can stabilize leaky blood vessels (140, 141). Preclinical studies have reported that these treatments can reprogram the immune microenvironment, for instance by converting immunosuppressive M2 macrophages to a pro-inflammatory M1 phenotype, further supporting antitumor immunity (142). Furthermore, CAR T cells can be genetically engineered to target components of the tumor vasculature, such as VEGFR2 or fibronectin splice variants like EIIIB, which may offer synergistic benefits of direct tumor cell killing and microenvironment remodeling (143, 144). Histological evidence supports that such decompression facilitates the infiltration of immune cells, including CAR T cells, into tumor tissue. Interestingly, the upregulation of superoxide dismutase 3 (SOD3) in the tumor microenvironment might be a mechanism to boost T cell infiltration by normalizing the tumor-associated endothelium (145, 146). Furthermore, it has been shown that combining CAR T therapy with oncolytic virotherapy not only induces tumor cell lysis, but also modifies the ECM and vasculature to facilitate better CAR T infiltration (147).

### Strategies to overcome the immunosuppressive TME

Another major challenge is the immunosuppressive TME which contributes to CAR T cell exhaustion and limits therapeutic efficacy. To overcome this barrier, various strategies have emerged to reprogram CAR T cells for enhanced persistence, activation, and resistance to TME-induced suppression. Targeting the immunosuppressive TME has yielded early clinical promise. For example, the infusion of TGF-β-resistant prostate-specific membrane antigen (PSMA)-targeting CAR T cells in metastatic castration-resistant prostate cancer achieved prostate-specific antigen (PSA) reduction in a subset of patients (148). However, CRS remains a dose-limiting toxicity, underscoring the need for safer designs.

To convert inhibitory signaling into productive immune responses, ‘switch’ CARs have been developed (149, 150). These synthetic receptors rewire suppressive signals from the TME into activating cues, and such designs are currently under evaluation in clinical trials (e.g. NCT06046040). Other engineering strategies involve equipping CAR T cells with the ability to secrete ICIs. For example, engineering CAR T cells to secrete PD-1-specific antibodies or knocking down immune checkpoint receptors, such as PD-1 and CTLA-4, enhances T cell function and reduces exhaustion (58, 151, 152). Trials investigating such constructs (e.g. NCT05089266, NCT05373147) aim to locally modulate the immunosuppressive TME, while avoiding systemic toxicities. Genetic modifications that delete or knock down checkpoint receptors during CAR T cell manufacturing are another promising avenue. PD-1 deletion has also been shown to enhance function, with clinical trials now evaluating such modifications (e.g. NCT05732948). Additionally, Good et al., showed improved CAR T cell killing in ID3 and SOX4 knock-out human CAR T cells demonstrates a role for these transcription factors in the dysfunction of CAR T cells (153).

‘Armored’ CAR T cells, which co-express pro-inflammatory cytokines, represent a powerful strategy to boost efficacy and persistence. CAR T cells have been engineered to express cytokines such as IL-7 and IL-21, which improve their expansion and persistence, and IL-23, which supports their proliferation and survival (154–156). In addition, cytokines like IL-12 and IL-36 help reshape the TME by promoting pro-inflammatory conditions favorable to CAR T cell activity (157, 158). IL-12, for instance, can activate endogenous cytotoxic T cells, NK cells, and polarize macrophages toward an anti-tumorigenic phenotype (159). Another critical advancement includes enhancing the trafficking and infiltration of CAR T cells into tumors by co-expressing IL-7 and the chemokine CCL19, thereby facilitating more effective tumor targeting (160). However, constitutive cytokine expression raises safety concerns, leading to the development of inducible expression systems or receptor-level engineering.

Metabolic reprogramming has also emerged as a promising approach. The metabolic constraints of the TME (hypoxia, nutrient deprivation, acidic pH, and oxidative stress) further limit CAR T cell functionality. Metabolic engineering strategies include developing hypoxia-responsive CARs that restrict expression to hypoxic regions, thereby enhancing safety and efficacy (161). Adjunct therapies, such as AMPK activators (e.g. metformin) and mTOR inhibitors (e.g. rapamycin), further support CAR T cell metabolic fitness (162). In this study, metformin and rapamycin pretreated CAR T cells demonstrated persistent and effective anti-glioma cytotoxic activities and significantly extended the survival of mice bearing intracerebral SB28 EGFRvIII gliomas. Importantly, human CAR T cells pretreated with metformin and rapamycin recapitulated the observations with murine CAR-T cells. These findings advocate for translational and clinical exploration of metformin and rapamycin pretreated CAR T cells in human trials. Furthermore, IL-10 (despite its conventional role as an immunosuppressive cytokine) can reprogram CAR T cell metabolism and restore their function in the exhausted state (163).

Enhancing endogenous immunity is another focus area; CAR T cells expressing Flt3 ligand (Flt3L) stimulate the expansion of DCs, boosting antigen presentation and the activation of the broader immune system (164). In terms of receptor engineering, constitutively active cytokine receptors and chimeric cytokine receptors are being incorporated into CAR T cells (165). These allow continuous signaling or convert inhibitory signals into activating ones, further supporting T cell survival and activity within suppressive environments. Additionally, integrating elements of the JAK-STAT signaling pathway into CAR constructs enhances CAR T proliferation and prevents premature differentiation (49). Orthogonal cytokine systems, such as IL-2 variants targeting synthetic receptors, are also under investigation enabling selective CAR T cell activation without triggering off-target immune responses (165, 166). To control these complex enhancements safely, researchers are developing synthetic gene circuits, such as the SynNotch platform, that allow precise regulation of therapeutic molecule expression (167).

## Combination therapies with CAR-engineered cells

**Figure 2:**
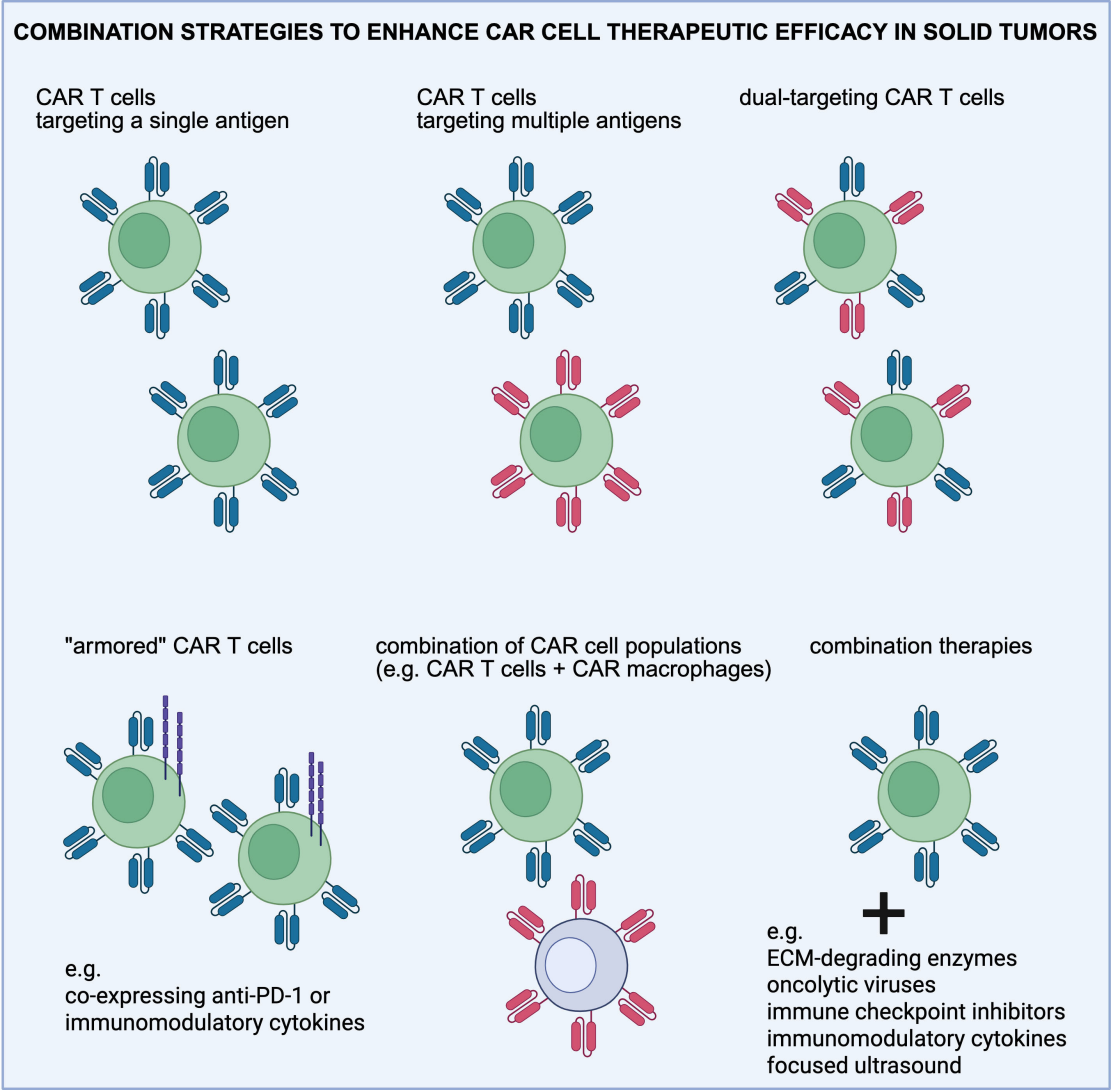
Overview of the combination strategies employed to enhance CAR T cell therapeutic efficacy in solid tumors. Combination treatments are aimed at targeting antigen heterogeneity, reprogramming the immunosuppressive tumor microenvironment, and normalizing the extracellular matrix (ECM). Created in https://BioRender.com.

CAR T cells have been engineered to secrete a diverse range of therapeutic payloads, including antibodies, enzymes, ICIs, and immunomodulatory proteins to directly modulate the TME and improve anti-tumor responses (**Figure 2**). Such combination treatments have shown an enhanced therapeutic effect on solid tumors. For example, mbIL12-engineered CAR T cells have been shown to promote durable anti-tumor responses against both regional and systemic disease in mice (168). This design led to increased IFNγ production, improved T cell proliferation, and recursive tumor cell killing *in vitro*. In addition, CAR T cells have been engineered to target the mesothelin antigen and secrete PD-L1 blocking antibody to remove the immunosuppressive effect of tumor on CAR T cells (termed Sec-MesoCAR-T cells) thereby increasing the therapeutic effect of CAR T cells on pancreatic cancer (169). Sec-MesoCAR-T cells showed an enhanced inhibitory effect on BxPC-3 tumor than MesoCAR-T cells *in vitro* and *in vivo*, and secreted higher level of cytokines including IL-2, IL-6 and IFN-*γ in vitro* than MesoCAR-T (control) cells. This work showed that the PD-L1 antibody secreted by Sec-MesoCAR-T cells relieved the immunosuppressive effect of pancreatic cancer on CAR T cells and improved their anti-tumor activity. Another group demonstrated that CAR T cells targeting tumor-specific epidermal growth factor receptor variant III (EGFRvIII) alone fail to control fully established tumors in an immunocompetent, orthotopic glioblastoma mouse model; however, when combined with a single, locally delivered dose of IL-12, achieve durable anti-tumor responses (170). These findings suggest that local delivery of IL-12 may be an effective adjuvant for CAR T cell therapy for glioblastoma. Furthermore, combination therapies incorporating CAR T cells with STING agonists are under exploration to modulate the TME and potentiate CAR T cell efficacy (171, 172). More recently, a focused ultrasound (FUS)-based approach was employed to acoustogenetically control the engineered T cells to transduce ultrasound signals into genetic and cellular activations for therapeutic applications *in vivo* (34). This acoustogenetics technology enables the activation of CAR T cells at confined tissue regions, allowing the targeting of less ideal antigens without causing non-specific off-tumor toxicity. Collectively, these efforts support the use of combination therapies together with CAR T cells for the treatment of solid tumors in the clinic.

## Conclusions

Recent clinical trials and patient experiences involving CAR T cell therapy for solid tumors have yielded promising results. While initial studies mostly focused on confirming safety, newer research has begun to demonstrate its potential effectiveness against complex and aggressive tumors. However, several obstacles still remain, such as difficulties in CAR T cell delivery, persistence, and tumor heterogeneity. Despite these challenges, innovative strategies are emerging. Targeting multiple tumor antigens at once is proving effective in addressing tumor variability and enhancing treatment outcomes. Recent advances, such as cytokine-armored T cells, may reduce the need for lymphodepleting chemotherapy before CAR T cell infusion. Additionally, combining CAR T cell therapy with oncolytic viruses, cytokines, and immune checkpoint inhibitors could help overcome tumor defenses. Insights from these studies are guiding the creation of more tailored and effective therapies. Enhancing the performance of CAR-engineered cells through ECM remodeling, metabolic reprogramming, and synthetic biology is expected to improve CAR T cell persistence, trafficking and cancer cell targeting, offering new hope for treating solid tumors successfully.

## References

[1] Patel KK Tariveranmoshabad M Kadu S Shobaki N June C. From concept to cure: The evolution of CAR-T cell therapy. Mol Ther. (2025) 33:2123–40. doi: 10.1016/j.ymthe.2025.03.005, PMID: 40070120 PMC12126787

[2] Curran KJ Brentjens RJ. Chimeric antigen receptor T cells for cancer immunotherapy. J Clin Oncol. (2015) 33:1703–6. doi: 10.1200/JCO.2014.60.3449, PMID: 25897155

[3] Maus MV Grupp SA Porter DL June CH. Antibody-modified T cells: CARs take the front seat for hematologic Malignancies. Blood. (2014) 123:2625–35. doi: 10.1182/blood-2013-11-492231, PMID: 24578504 PMC3999751

[4] Uslu U June CH. Beyond the blood: expanding CAR T cell therapy to solid tumors. Nat Biotechnol. (2025) 43:506–15. doi: 10.1038/s41587-024-02446-2, PMID: 39533105

[5] Du B Qin J Lin B Zhang J Li D Liu M. CAR-T therapy in solid tumors. Cancer Cell. (2025) 43:665–79. doi: 10.1016/j.ccell.2025.03.019, PMID: 40233718

[6] Maalej KM Merhi M Inchakalody VP Mestiri S Alam M Maccalli C. CAR-cell therapy in the era of solid tumor treatment: current challenges and emerging therapeutic advances. Mol Cancer. (2023) 22:20. doi: 10.1186/s12943-023-01723-z, PMID: 36717905 PMC9885707

[7] Maude SL Laetsch TW Buechner J Rives S Boyer M Bittencourt H. Tisagenlecleucel in children and young adults with B-cell lymphoblastic leukemia. N Engl J Med. (2018) 378:439–48. doi: 10.1056/NEJMoa1709866, PMID: 29385370 PMC5996391

[8] Neelapu SS Locke FL Bartlett NL Lekakis LJ Miklos DB Jacobson CA. Axicabtagene ciloleucel CAR T-cell therapy in refractory large B-cell lymphoma. N Engl J Med. (2017) 377:2531–44. doi: 10.1056/NEJMoa1707447, PMID: 29226797 PMC5882485

[9] Ahmed N Brawley VS Hegde M Robertson C Ghazi A Gerken C. Human epidermal growth factor receptor 2 (HER2) -specific chimeric antigen receptor-modified T cells for the immunotherapy of HER2-positive sarcoma. J Clin Oncol. (2015) 33:1688–96. doi: 10.1200/JCO.2014.58.0225, PMID: 25800760 PMC4429176

[10] Chen N Pu C Zhao L Li W Wang C Zhu R. Chimeric antigen receptor T cells targeting CD19 and GCC in metastatic colorectal cancer: A nonrandomized clinical trial. JAMA Oncol. (2024) 10:1532–6. doi: 10.1001/jamaoncol.2024.3891, PMID: 39298141 PMC11413756

[11] Spiegel JY Patel S Muffly L Hossain NM Oak J Baird JH. CAR T cells with dual targeting of CD19 and CD22 in adult patients with recurrent or refractory B cell Malignancies: a phase 1 trial. Nat Med. (2021) 27:1419–31. doi: 10.1038/s41591-021-01436-0, PMID: 34312556 PMC8363505

[12] Liu Y Guo Y Wu Z Feng K Tong C Wang Y. Anti-EGFR chimeric antigen receptor-modified T cells in metastatic pancreatic carcinoma: A phase I clinical trial. Cytotherapy. (2020) 22:573–80. doi: 10.1016/j.jcyt.2020.04.088, PMID: 32527643

[13] Wang Y Chen M Wu Z Tong C Dai H Guo Y. CD133-directed CAR T cells for advanced metastasis Malignancies: A phase I trial. Oncoimmunology. (2018) 7:e1440169. doi: 10.1080/2162402X.2018.1440169, PMID: 29900044 PMC5993480

[14] Munshi NC Anderson LD Jr. Shah N Madduri D Berdeja J Lonial S. Idecabtagene vicleucel in relapsed and refractory multiple myeloma. N Engl J Med. (2021) 384:705–16. doi: 10.1056/NEJMoa2024850, PMID: 33626253

[15] Berdeja JG Madduri D Usmani SZ Jakubowiak A Agha M Cohen AD. Ciltacabtagene autoleucel, a B-cell maturation antigen-directed chimeric antigen receptor T-cell therapy in patients with relapsed or refractory multiple myeloma (CARTITUDE-1): a phase 1b/2 open-label study. Lancet. (2021) 398:314–24. doi: 10.1016/S0140-6736(21)00933-8, PMID: 34175021

[16] Botta GP Becerra CR Jin Z Kim DW Zhao D Lenz H-J. Multicenter phase Ib trial in the U.S. @ of salvage CT041 CLDN18.2-specific chimeric antigen receptor T-cell therapy for patients with advanced gastric and pancreatic adenocarcinoma. J Clin Oncol. (2022) 20:2538. doi: 10.1200/JCO.2022.40.16_suppl.2538

[17] Feng K Liu Y Guo Y Qiu J Wu Z Dai H. Phase I study of chimeric antigen receptor modified T cells in treating HER2-positive advanced biliary tract cancers and pancreatic cancers. Protein Cell. (2018) 9:838–47. doi: 10.1007/s13238-017-0440-4, PMID: 28710747 PMC6160389

[18] Li CH Sharma S Heczey AA Woods ML Steffin DHM Louis CU. Long-term outcomes of GD2-directed CAR-T cell therapy in patients with neuroblastoma. Nat Med. (2025) 31:1125–9. doi: 10.1038/s41591-025-03513-0, PMID: 39962287

[19] Haas AR Tanyi JL O’Hara MH Gladney WL Lacey SF Torigian DA. Phase I study of lentiviral-transduced chimeric antigen receptor-modified T cells recognizing mesothelin in advanced solid cancers. Mol Ther. (2019) 27:1919–29. doi: 10.1016/j.ymthe.2019.07.015, PMID: 31420241 PMC6838875

[20] Kimman T Cuenca M Tieland RG Rockx-Brouwer D Janssen J Motais B. Engineering anti-BCMA CAR T cells for enhancing myeloma killing efficacy via apoptosis regulation. Nat Commun. (2025) 16:4638. doi: 10.1038/s41467-025-59818-8, PMID: 40389394 PMC12089368

[21] Yao H Ren SH Wang LH Ren MQ Cai J Chen D. BCMA/GPRC5D bispecific CAR T-cell therapy for relapsed/refractory multiple myeloma with extramedullary disease: a single-center, single-arm, phase 1 trial. J Hematol Oncol. (2025) 18:56. doi: 10.1186/s13045-025-01713-2, PMID: 40383818 PMC12087025

[22] Del Bufalo F De Angelis B Caruana I Del Baldo G De Ioris MA Serra A. GD2-CART01 for relapsed or refractory high-risk neuroblastoma. N Engl J Med. (2023) 388:1284–95. doi: 10.1056/NEJMoa2210859, PMID: 37018492

[23] Newick K O'Brien S Moon E Albelda SM. CAR T cell therapy for solid tumors. Annu Rev Med. (2017) 68:139–52. doi: 10.1146/annurev-med-062315-120245, PMID: 27860544

[24] Liu E Marin D Banerjee P Macapinlac HA Thompson P Basar R. Use of CAR-transduced natural killer cells in CD19-positive lymphoid tumors. N Engl J Med. (2020) 382:545–53. doi: 10.1056/NEJMoa1910607, PMID: 32023374 PMC7101242

[25] Deuse T Schrepfer S. Progress and challenges in developing allogeneic cell therapies. Cell Stem Cell. (2025) 32:513–28. doi: 10.1016/j.stem.2025.03.004, PMID: 40185072

[26] Posey AD Jr. Young RM June CH. Future perspectives on engineered T cells for cancer. Trends Cancer. (2024) 10:687–95. doi: 10.1016/j.trecan.2024.05.007, PMID: 38853073

[27] Lee DW Gardner R Porter DL Louis CU Ahmed N Jensen M. Current concepts in the diagnosis and management of cytokine release syndrome. Blood. (2014) 124:188–95. doi: 10.1182/blood-2014-05-552729, PMID: 24876563 PMC4093680

[28] Amatya C Pegues MA Lam N Vanasse D Geldres C Choi S. Development of CAR T cells expressing a suicide gene plus a chimeric antigen receptor targeting signaling lymphocytic-activation molecule F7. Mol Ther. (2021) 29:702–17. doi: 10.1016/j.ymthe.2020.10.008, PMID: 33129371 PMC7854354

[29] Bouquet L Bole-Richard E Warda W Neto Da Rocha M Trad R Nicod C. RapaCaspase-9-based suicide gene applied to the safety of IL-1RAP CAR-T cells. Gene Ther. (2023) 30:706–13. doi: 10.1038/s41434-023-00404-2, PMID: 37173386 PMC10506905

[30] Locke FL Miklos DB Jacobson CA Perales MA Kersten MJ Oluwole OO. Axicabtagene ciloleucel as second-line therapy for large B-cell lymphoma. N Engl J Med. (2022) 386:640–54. doi: 10.1056/NEJMoa2116133, PMID: 34891224

[31] Liu Y Zheng Y Deng T Huang Y Liu Z Zhan B. Oncolytic herpes simplex virus delivery of dual CAR targets of CD19 and BCMA as well as immunomodulators to enhance therapeutic efficacy in solid tumors combined with CAR T cell therapy. Front Oncol. (2022) 12:1037934. doi: 10.3389/fonc.2022.1037934, PMID: 36353540 PMC9638445

[32] Satapathy BP Sheoran P Yadav R Chettri D Sonowal D Dash CP. The synergistic immunotherapeutic impact of engineered CAR-T cells with PD-1 blockade in lymphomas and solid tumors: a systematic review. Front Immunol. (2024) 15:1389971. doi: 10.3389/fimmu.2024.1389971, PMID: 38799440 PMC11116574

[33] Stock S Kluever AK Endres S Kobold S. Enhanced chimeric antigen receptor T cell therapy through co-application of synergistic combination partners. Biomedicines. (2022) 10:307. doi: 10.3390/biomedicines10020307, PMID: 35203517 PMC8869718

[34] Zhang W Huang C Liu R Zhang H Li W Yin S. Case report: CD19-directed CAR-T cell therapy combined with BTK inhibitor and PD-1 antibody against secondary central nervous system lymphoma. Front Immunol. (2022) 13:983934. doi: 10.3389/fimmu.2022.983934, PMID: 36275715 PMC9581047

[35] Maloney DG Kuruvilla J Liu FF Kostic A Kim Y Bonner A. Matching-adjusted indirect treatment comparison of liso-cel versus axi-cel in relapsed or refractory large B cell lymphoma. J Hematol Oncol. (2021) 14:140. doi: 10.1186/s13045-021-01144-9, PMID: 34493319 PMC8425084

[36] Melenhorst JJ Chen GM Wang M Porter DL Chen C Collins MA. Decade-long leukaemia remissions with persistence of CD4(+) CAR T cells. Nature. (2022) 602:503–9. doi: 10.1038/s41586-021-04390-6, PMID: 35110735 PMC9166916

[37] Sommermeyer D Hudecek M Kosasih PL Gogishvili T Maloney DG Turtle CJ. Chimeric antigen receptor-modified T cells derived from defined CD8+ and CD4+ subsets confer superior antitumor reactivity in *vivo* . Leukemia. (2016) 30:492–500. doi: 10.1038/leu.2015.247, PMID: 26369987 PMC4746098

[38] Friedman KM Garrett TE Evans JW Horton HM Latimer HJ Seidel SL. Effective targeting of multiple B-cell maturation antigen-expressing hematological Malignances by anti-B-cell maturation antigen chimeric antigen receptor T cells. Hum Gene Ther. (2018) 29:585–601. doi: 10.1089/hum.2018.001, PMID: 29641319 PMC5930946

[39] Pan K Farrukh H Chittepu V Xu H Pan CX Zhu Z. CAR race to cancer immunotherapy: from CAR T, CAR NK to CAR macrophage therapy. J Exp Clin Cancer Res. (2022) 41:119. doi: 10.1186/s13046-022-02327-z, PMID: 35361234 PMC8969382

[40] Peng L Sferruzza G Yang L Zhou L Chen S. CAR-T and CAR-NK as cellular cancer immunotherapy for solid tumors. Cell Mol Immunol. (2024) 21:1089–108. doi: 10.1038/s41423-024-01207-0, PMID: 39134804 PMC11442786

[41] Chen J Qiu S Li W Wang K Zhang Y Yang H. Tuning charge density of chimeric antigen receptor optimizes tonic signaling and CAR-T cell fitness. Cell Res. (2023) 33:341–54. doi: 10.1038/s41422-023-00789-0, PMID: 36882513 PMC10156745

[42] Li W Qiu S Chen J Jiang S Chen W Jiang J. Chimeric antigen receptor designed to prevent ubiquitination and downregulation showed durable antitumor efficacy. Immunity. (2020) 53:456–470 e456. doi: 10.1016/j.immuni.2020.07.011, PMID: 32758419

[43] Roddie C Lekakis LJ Marzolini MAV Ramakrishnan A Zhang Y Hu Y. Dual targeting of CD19 and CD22 with bicistronic CAR-T cells in patients with relapsed/refractory large B-cell lymphoma. Blood. (2023) 141:2470–82. doi: 10.1182/blood.2022018598, PMID: 36821767 PMC10646794

[44] Wei J Long L Zheng W Dhungana Y Lim SA Guy C. Targeting REGNASE-1 programs long-lived effector T cells for cancer therapy. Nature. (2019) 576:471–6. doi: 10.1038/s41586-019-1821-z, PMID: 31827283 PMC6937596

[45] Kowolik CM Topp MS Gonzalez S Pfeiffer T Olivares S Gonzalez N. CD28 costimulation provided through a CD19-specific chimeric antigen receptor enhances *in vivo* persistence and antitumor efficacy of adoptively transferred T cells. Cancer Res. (2006) 66:10995–1004. doi: 10.1158/0008-5472.CAN-06-0160, PMID: 17108138

[46] Kalos M Levine BL Porter DL Katz S Grupp SA Bagg A. T cells with chimeric antigen receptors have potent antitumor effects and can establish memory in patients with advanced leukemia. Sci Transl Med. (2011) 3:95ra73. doi: 10.1126/scitranslmed.3002842, PMID: 21832238 PMC3393096

[47] Corti C Venetis K Sajjadi E Zattoni L Curigliano G Fusco N. CAR-T cell therapy for triple-negative breast cancer and other solid tumors: preclinical and clinical progress. Expert Opin Investig Drugs. (2022) 31:593–605. doi: 10.1080/13543784.2022.2054326, PMID: 35311430

[48] Martinez-Lostao L Anel A Pardo J. How do cytotoxic lymphocytes kill cancer cells? Clin Cancer Res. (2015) 21:5047–56. doi: 10.1158/1078-0432.CCR-15-0685, PMID: 26567364

[49] Kagoya Y Tanaka S Guo T Anczurowski M Wang CH Saso K. A novel chimeric antigen receptor containing a JAK-STAT signaling domain mediates superior antitumor effects. Nat Med. (2018) 24:352–9. doi: 10.1038/nm.4478, PMID: 29400710 PMC5839992

[50] Hegde M Corder A Chow KK Mukherjee M Ashoori A Kew Y. Combinational targeting offsets antigen escape and enhances effector functions of adoptively transferred T cells in glioblastoma. Mol Ther. (2013) 21:2087–101. doi: 10.1038/mt.2013.185, PMID: 23939024 PMC3831041

[51] Naghizadeh A Tsao WC Hyun Cho J Xu H Mohamed M Li D. *In vitro* machine learning-based CAR T immunological synapse quality measurements correlate with patient clinical outcomes. PloS Comput Biol. (2022) 18:e1009883. doi: 10.1371/journal.pcbi.1009883, PMID: 35303007 PMC8955962

[52] Vercellino L de Jong D di Blasi R Kanoun S Reshef R Schwartz LH. Current and future role of medical imaging in guiding the management of patients with relapsed and refractory non-hodgkin lymphoma treated with CAR T-cell therapy. Front Oncol. (2021) 11:664688. doi: 10.3389/fonc.2021.664688, PMID: 34123825 PMC8195284

[53] Kershaw MH Wang G Westwood JA Pachynski RK Tiffany HL Marincola FM. Redirecting migration of T cells to chemokine secreted from tumors by genetic modification with CXCR2. Hum Gene Ther. (2002) 13:1971–80. doi: 10.1089/10430340260355374, PMID: 12427307

[54] Thadi A Khalili M Morano WF Richard SD Katz SC Bowne WB. Early investigations and recent advances in intraperitoneal immunotherapy for peritoneal metastasis. Vaccines (Basel). (2018) 6:54. doi: 10.3390/vaccines6030054, PMID: 30103457 PMC6160982

[55] Li H Wang Z Ogunnaike EA Wu Q Chen G Hu Q. Scattered seeding of CAR T cells in solid tumors augments anticancer efficacy. Natl Sci Rev. (2022) 9:nwab172. doi: 10.1093/nsr/nwab172, PMID: 35265340 PMC8900686

[56] Zheng R Shen K Liang S Lyu Y Zhang S Dong H. Specific ECM degradation potentiates the antitumor activity of CAR-T cells in solid tumors. Cell Mol Immunol. (2024) 21:1491–504. doi: 10.1038/s41423-024-01228-9, PMID: 39472748 PMC11606952

[57] Cherkassky L Morello A Villena-Vargas J Feng Y Dimitrov DS Jones DR. Human CAR T cells with cell-intrinsic PD-1 checkpoint blockade resist tumor-mediated inhibition. J Clin Invest. (2016) 126:3130–44. doi: 10.1172/JCI83092, PMID: 27454297 PMC4966328

[58] Rafiq S Yeku OO Jackson HJ Purdon TJ van Leeuwen DG Drakes DJ. Targeted delivery of a PD-1-blocking scFv by CAR-T cells enhances anti-tumor efficacy in *vivo* . Nat Biotechnol. (2018) 36:847–56. doi: 10.1038/nbt.4195, PMID: 30102295 PMC6126939

[59] Yoon DH Osborn MJ Tolar J Kim CJ. Incorporation of immune checkpoint blockade into chimeric antigen receptor T cells (CAR-ts): combination or built-in CAR-T. Int J Mol Sci. (2018) 19:340. doi: 10.3390/ijms19020340, PMID: 29364163 PMC5855562

[60] Northcott JM Dean IS Mouw JK Weaver VM. Feeling stress: the mechanics of cancer progression and aggression. Front Cell Dev Biol. (2018) 6:17. doi: 10.3389/fcell.2018.00017, PMID: 29541636 PMC5835517

[61] Kalli M Stylianopoulos T. Defining the role of solid stress and matrix stiffness in cancer cell proliferation and metastasis. Front Oncol. (2018) 8:55. doi: 10.3389/fonc.2018.00055, PMID: 29594037 PMC5857934

[62] Stylianopoulos T Martin JD Snuderl M Mpekris F Jain SR Jain RK. Coevolution of solid stress and interstitial fluid pressure in tumors during progression: implications for vascular collapse. Cancer Res. (2013) 73:3833–41. doi: 10.1158/0008-5472.CAN-12-4521, PMID: 23633490 PMC3702668

[63] Voutouri C Stylianopoulos T. Accumulation of mechanical forces in tumors is related to hyaluronan content and tissue stiffness. PloS One. (2018) 13:e0193801. doi: 10.1371/journal.pone.0193801, PMID: 29561855 PMC5862434

[64] Kalli M Papageorgis P Gkretsi V Stylianopoulos T. Solid stress facilitates fibroblasts activation to promote pancreatic cancer cell migration. Ann BioMed Eng. (2018) 46:657–69. doi: 10.1007/s10439-018-1997-7, PMID: 29470747 PMC5951267

[65] Linke JA Munn LL Jain RK. Compressive stresses in cancer: characterization and implications for tumour progression and treatment. Nat Rev Cancer. (2024) 24:768–91. doi: 10.1038/s41568-024-00745-z, PMID: 39390249 PMC12967324

[66] Nia HT Liu H Seano G Datta M Jones D Rahbari N. Solid stress and elastic energy as measures of tumour mechanopathology. Nat BioMed Eng. (2016) 1:0004. doi: 10.1038/s41551-016-0004, PMID: 28966873 PMC5621647

[67] Kalli M Poskus MD Stylianopoulos T Zervantonakis IK. Beyond matrix stiffness: targeting force-induced cancer drug resistance. Trends Cancer. (2023) 9:937–54. doi: 10.1016/j.trecan.2023.07.006, PMID: 37558577 PMC10592424

[68] Kalli M Stylianopoulos T. Toward innovative approaches for exploring the mechanically regulated tumor-immune microenvironment. APL Bioeng. (2024) 8:011501. doi: 10.1063/5.0183302, PMID: 38390314 PMC10883717

[69] Jain RK Martin JD Stylianopoulos T. The role of mechanical forces in tumor growth and therapy. Annu Rev BioMed Eng. (2014) 16:321–46. doi: 10.1146/annurev-bioeng-071813-105259, PMID: 25014786 PMC4109025

[70] Mpekris F Panagi M Charalambous A Voutouri C Stylianopoulos T. Modulating cancer mechanopathology to restore vascular function and enhance immunotherapy. Cell Rep Med. (2024) 5:101626. doi: 10.1016/j.xcrm.2024.101626, PMID: 38944037 PMC11293360

[71] Stylianopoulos T Munn LL Jain RK. Reengineering the physical microenvironment of tumors to improve drug delivery and efficacy: from mathematical modeling to bench to bedside. Trends Cancer. (2018) 4:292–319. doi: 10.1016/j.trecan.2018.02.005, PMID: 29606314 PMC5930008

[72] Nia HT Munn LL Jain RK. Physical traits of cancer. Science. (2020) 370(6516). doi: 10.1126/science.aaz0868, PMID: 33122355 PMC8274378

[73] Fukumura D Kloepper J Amoozgar Z Duda DG Jain RK. Enhancing cancer immunotherapy using antiangiogenics: opportunities and challenges. Nat Rev Clin Oncol. (2018) 15:325–40. doi: 10.1038/nrclinonc.2018.29, PMID: 29508855 PMC5921900

[74] Chen W Zhu C. Mechanical regulation of T-cell functions. Immunol Rev. (2013) 256:160–76. doi: 10.1111/imr.12122, PMID: 24117820 PMC3818107

[75] Al-Aghbar MA Jainarayanan AK Dustin ML Roffler SR. The interplay between membrane topology and mechanical forces in regulating T cell receptor activity. Commun Biol. (2022) 5:40. doi: 10.1038/s42003-021-02995-1, PMID: 35017678 PMC8752658

[76] Li YC Chen BM Wu PC Cheng TL Kao LS Tao MH. Cutting Edge: mechanical forces acting on T cells immobilized via the TCR complex can trigger TCR signaling. J Immunol. (2010) 184:5959–63. doi: 10.4049/jimmunol.0900775, PMID: 20435924

[77] Meng KP Majedi FS Thauland TJ Butte MJ. Mechanosensing through YAP controls T cell activation and metabolism. J Exp Med. (2020) 217(8):e20200053. doi: 10.1084/jem.20200053, PMID: 32484502 PMC7398163

[78] Bollyky PL Falk BA Wu RP Buckner JH Wight TN Nepom GT. Intact extracellular matrix and the maintenance of immune tolerance: high molecular weight hyaluronan promotes persistence of induced CD4+CD25+ regulatory T cells. J Leukoc Biol. (2009) 86:567–72. doi: 10.1189/jlb.0109001, PMID: 19401397 PMC2735281

[79] Wan Z Xu C Chen X Xie H Li Z Wang J. PI(4,5)P2 determines the threshold of mechanical force-induced B cell activation. J Cell Biol. (2018) 217:2565–82. doi: 10.1083/jcb.201711055, PMID: 29685902 PMC6028545

[80] Shan S Fang B Zhang Y Wang C Zhou J Niu C. Mechanical stretch promotes tumoricidal M1 polarization via the FAK/NF-kappaB signaling pathway. FASEB J. (2019) 33:13254–66. doi: 10.1096/fj.201900799RR, PMID: 31539281

[81] Atcha H Jairaman A Holt JR Meli VS Nagalla RR Veerasubramanian PK. Mechanically activated ion channel Piezo1 modulates macrophage polarization and stiffness sensing. Nat Commun. (2021) 12:3256. doi: 10.1038/s41467-021-23482-5, PMID: 34059671 PMC8167181

[82] Meli VS Atcha H Veerasubramanian PK Nagalla RR Luu TU Chen EY. YAP-mediated mechanotransduction tunes the macrophage inflammatory response. Sci Adv. (2020) 6:eabb8471. doi: 10.1126/sciadv.abb8471, PMID: 33277245 PMC7717914

[83] Maller O Drain AP Barrett AS Borgquist S Ruffell B Zakharevich I. Tumour-associated macrophages drive stromal cell-dependent collagen crosslinking and stiffening to promote breast cancer aggression. Nat Mater. (2021) 20:548–59. doi: 10.1038/s41563-020-00849-5, PMID: 33257795 PMC8005404

[84] Li R Serrano JC Xing H Lee TA Azizgolshani H Zaman M. Interstitial flow promotes macrophage polarization toward an M2 phenotype. Mol Biol Cell. (2018) 29:1927–40. doi: 10.1091/mbc.E18-03-0164, PMID: 29995595 PMC6232969

[85] Solis AG Bielecki P Steach HR Sharma L Harman CCD Yun S. Mechanosensation of cyclical force by PIEZO1 is essential for innate immunity. Nature. (2019) 573:69–74. doi: 10.1038/s41586-019-1485-8, PMID: 31435009 PMC6939392

[86] Dombroski JA Rowland SJ Fabiano AR Knoblauch SV Hope JM King MR. Fluid shear stress enhances dendritic cell activation. Immunobiology. (2023) 228:152744. doi: 10.1016/j.imbio.2023.152744, PMID: 37729773 PMC10841200

[87] Kang JH Lee HJ Kim OH Yun YJ Seo YJ Lee HJ. Biomechanical forces enhance directed migration and activation of bone marrow-derived dendritic cells. Sci Rep. (2021) 11:12106. doi: 10.1038/s41598-021-91117-2, PMID: 34103554 PMC8187447

[88] Wu J Wu D Wu G Bei HP Li Z Xu H. Scale-out production of extracellular vesicles derived from natural killer cells via mechanical stimulation in a seesaw-motion bioreactor for cancer therapy. Biofabrication. (2022) 14(4). doi: 10.1088/1758-5090/ac7eeb, PMID: 35793612

[89] Brand A Singer K Koehl GE Kolitzus M Schoenhammer G Thiel A. LDHA-associated lactic acid production blunts tumor immunosurveillance by T and NK cells. Cell Metab. (2016) 24:657–71. doi: 10.1016/j.cmet.2016.08.011, PMID: 27641098

[90] Fischer K Hoffmann P Voelkl S Meidenbauer N Ammer J Edinger M. Inhibitory effect of tumor cell-derived lactic acid on human T cells. Blood. (2007) 109:3812–9. doi: 10.1182/blood-2006-07-035972, PMID: 17255361

[91] Parks SK Cormerais Y Marchiq I Pouyssegur J. Hypoxia optimises tumour growth by controlling nutrient import and acidic metabolite export. Mol Aspects Med. (2016) 47-48:3–14. doi: 10.1016/j.mam.2015.12.001, PMID: 26724171

[92] Zhang L Yu D. Exosomes in cancer development, metastasis, and immunity. Biochim Biophys Acta Rev Cancer. (2019) 1871:455–68. doi: 10.1016/j.bbcan.2019.04.004, PMID: 31047959 PMC6542596

[93] Gunderson AJ Yamazaki T McCarty K Fox N Phillips M Alice A. TGFbeta suppresses CD8(+) T cell expression of CXCR3 and tumor trafficking. Nat Commun. (2020) 11:1749. doi: 10.1038/s41467-020-15404-8, PMID: 32273499 PMC7145847

[94] Lohr J Ratliff T Huppertz A Ge Y Dictus C Ahmadi R. Effector T-cell infiltration positively impacts survival of glioblastoma patients and is impaired by tumor-derived TGF-beta. Clin Cancer Res. (2011) 17:4296–308. doi: 10.1158/1078-0432.CCR-10-2557, PMID: 21478334

[95] Mariathasan S Turley SJ Nickles D Castiglioni A Yuen K Wang Y. TGFbeta attenuates tumour response to PD-L1 blockade by contributing to exclusion of T cells. Nature. (2018) 554:544–8. doi: 10.1038/nature25501, PMID: 29443960 PMC6028240

[96] Flavell RA Sanjabi S Wrzesinski SH Licona-Limon P. The polarization of immune cells in the tumour environment by TGFbeta. Nat Rev Immunol. (2010) 10:554–67. doi: 10.1038/nri2808, PMID: 20616810 PMC3885992

[97] Driemel C Kremling H Schumacher S Will D Wolters J Lindenlauf N. Context-dependent adaption of EpCAM expression in early systemic esophageal cancer. Oncogene. (2014) 33:4904–15. doi: 10.1038/onc.2013.441, PMID: 24141784

[98] Tan W Zhang W Strasner A Grivennikov S Cheng JQ Hoffman RM. Tumour-infiltrating regulatory T cells stimulate mammary cancer metastasis through RANKL-RANK signalling. Nature. (2011) 470:548–53. doi: 10.1038/nature09707, PMID: 21326202 PMC3166217

[99] Swartz MA Lund AW. Lymphatic and interstitial flow in the tumour microenvironment: linking mechanobiology with immunity. Nat Rev Cancer. (2012) 12:210–9. doi: 10.1038/nrc3186, PMID: 22362216

[100] Liu Y Zhang T Zhang H Li J Zhou N Fiskesund R. Cell softness prevents cytolytic T-cell killing of tumor-repopulating cells. Cancer Res. (2021) 81:476–88. doi: 10.1158/0008-5472.CAN-20-2569, PMID: 33168645

[101] Fucikova J Moserova I Truxova I Hermanova I Vancurova I Partlova S. High hydrostatic pressure induces immunogenic cell death in human tumor cells. Int J Cancer. (2014) 135:1165–77. doi: 10.1002/ijc.28766, PMID: 24500981

[102] Wang Y Goliwas KF Severino PE Hough KP Van Vessem D Wang H. Mechanical strain induces phenotypic changes in breast cancer cells and promotes immunosuppression in the tumor microenvironment. Lab Invest. (2020) 100:1503–16. doi: 10.1038/s41374-020-0452-1, PMID: 32572176 PMC7686122

[103] Baginska J Viry E Berchem G Poli A Noman MZ van Moer K. Granzyme B degradation by autophagy decreases tumor cell susceptibility to natural killer-mediated lysis under hypoxia. Proc Natl Acad Sci U.S.A. (2013) 110:17450–5. doi: 10.1073/pnas.1304790110, PMID: 24101526 PMC3808626

[104] Kalli M Mpekris F Charalambous A Michael C Stylianou C Voutouri C. Mechanical forces inducing oxaliplatin resistance in pancreatic cancer can be targeted by autophagy inhibition. Commun Biol. (2024) 7:1581. doi: 10.1038/s42003-024-07268-1, PMID: 39604540 PMC11603328

[105] Yamamoto K Venida A Yano J Biancur DE Kakiuchi M Gupta S. Autophagy promotes immune evasion of pancreatic cancer by degrading MHC-I. Nature. (2020) 581:100–5. doi: 10.1038/s41586-020-2229-5, PMID: 32376951 PMC7296553

[106] Wang H Yao H Li C Shi H Lan J Li Z. HIP1R targets PD-L1 to lysosomal degradation to alter T cell-mediated cytotoxicity. Nat Chem Biol. (2019) 15:42–50. doi: 10.1038/s41589-018-0161-x, PMID: 30397328

[107] Nair R Somasundaram V Kuriakose A Krishn SR Raben D Salazar R. Deciphering T-cell exhaustion in the tumor microenvironment: paving the way for innovative solid tumor therapies. Front Immunol. (2025) 16:1548234. doi: 10.3389/fimmu.2025.1548234, PMID: 40236693 PMC11996672

[108] Garces-Lazaro I Kotzur R Cerwenka A Mandelboim O. NK cells under hypoxia: the two faces of vascularization in tumor and pregnancy. Front Immunol. (2022) 13:924775. doi: 10.3389/fimmu.2022.924775, PMID: 35769460 PMC9234265

[109] Kennedy PR Arvindam US Phung SK Ettestad B Feng X Li Y. Metabolic programs drive function of therapeutic NK cells in hypoxic tumor environments. Sci Adv. (2024) 10:eadn1849. doi: 10.1126/sciadv.adn1849, PMID: 39475618 PMC11524192

[110] Zhao R Zhou X Khan ES Alansary D Friedmann KS Yang W. Targeting the microtubule-network rescues CTL killing efficiency in dense 3D matrices. Front Immunol. (2021) 12:729820. doi: 10.3389/fimmu.2021.729820, PMID: 34484240 PMC8416057

[111] Zhang J Li J Hou Y Lin Y Zhao H Shi Y. Osr2 functions as a biomechanical checkpoint to aggravate CD8(+) T cell exhaustion in tumor. Cell. (2024) 187:3409–3426 e3424. doi: 10.1016/j.cell.2024.04.023, PMID: 38744281

[112] Perez Del Rio E Santos F Rodriguez Rodriguez X Martinez-Miguel M Roca-Pinilla R Aris A. CCL21-loaded 3D hydrogels for T cell expansion and differentiation. Biomaterials. (2020) 259:120313. doi: 10.1016/j.biomaterials.2020.120313, PMID: 32829146

[113] Atik AF Suryadevara CM Schweller RM West JL Healy P Herndon Ii JE. Hyaluronic acid based low viscosity hydrogel as a novel carrier for Convection Enhanced Delivery of CAR T cells. J Clin Neurosci. (2018) 56:163–8. doi: 10.1016/j.jocn.2018.06.005, PMID: 30041899 PMC6185757

[114] Liu L Qu Y Cheng L Yoon CW He P Monther A. Engineering chimeric antigen receptor T cells for solid tumour therapy. Clin Transl Med. (2022) 12:e1141. doi: 10.1002/ctm2.1141, PMID: 36495108 PMC9736813

[115] Caruana I Savoldo B Hoyos V Weber G Liu H Kim ES. Heparanase promotes tumor infiltration and antitumor activity of CAR-redirected T lymphocytes. Nat Med. (2015) 21:524–9. doi: 10.1038/nm.3833, PMID: 25849134 PMC4425589

[116] Martin-Otal C Lasarte-Cia A Serrano D Casares N Conde E Navarro F. Targeting the extra domain A of fibronectin for cancer therapy with CAR-T cells. J Immunother Cancer. (2022) 10(8):e004479. doi: 10.1136/jitc-2021-004479, PMID: 35918123 PMC9351345

[117] Nota S Osei-Hwedieh DO Drum DL Wang X Sabbatino F Ferrone S. Chondroitin sulfate proteoglycan 4 expression in chondrosarcoma: A potential target for antibody-based immunotherapy. Front Oncol. (2022) 12:939166. doi: 10.3389/fonc.2022.939166, PMID: 36110930 PMC9468862

[118] Wickman E Lange S Wagner J Ibanez J Tian L Lu M. IL-18R supported CAR T cells targeting oncofetal tenascin C for the immunotherapy of pediatric sarcoma and brain tumors. J Immunother Cancer. (2024) 12(11):e009743. doi: 10.1136/jitc-2024-009743, PMID: 39572158 PMC11580246

[119] Yassin AA Urena Martin C Le Saux G Pandey A Tzadka S Toledo E. Mechanostimulatory platform for improved CAR T cell immunotherapy. Adv Mater. (2025) 37:e2412482. doi: 10.1002/adma.202412482, PMID: 40348587

[120] Li HR Ye BC. Engineered probiotic-mediated intratumoral delivery and controlled release of bacterial collagenase for cancer therapy. Synth Syst Biotechnol. (2025) 10:226–36. doi: 10.1016/j.synbio.2024.09.001, PMID: 39582690 PMC11585803

[121] Zhang Y Pei P Zhou H Xie Y Yang S Shen W. Nattokinase-mediated regulation of tumor physical microenvironment to enhance chemotherapy, radiotherapy, and CAR-T therapy of solid tumor. ACS Nano. (2023) 17:7475–86. doi: 10.1021/acsnano.2c12463, PMID: 37057972

[122] Yang XY Zhang JG Zhou QM Yu JN Lu YF Wang XJ. Extracellular matrix modulating enzyme functionalized biomimetic Au nanoplatform-mediated enhanced tumor penetration and synergistic antitumor therapy for pancreatic cancer. J Nanobiotechnology. (2022) 20:524. doi: 10.1186/s12951-022-01738-6, PMID: 36496411 PMC9741808

[123] Jain RK. Normalizing tumor microenvironment to treat cancer: bench to bedside to biomarkers. J Clin Oncol. (2013) 31:2205–18. doi: 10.1200/JCO.2012.46.3653, PMID: 23669226 PMC3731977

[124] Mpekris F Panagi M Charalambous A Voutouri C Michael C Papoui A. A synergistic approach for modulating the tumor microenvironment to enhance nano-immunotherapy in sarcomas. Neoplasia. (2024) 51:100990. doi: 10.1016/j.neo.2024.100990, PMID: 38520790 PMC10978543

[125] Panagi M Mpekris F Voutouri C Hadjigeorgiou AG Symeonidou C Porfyriou E. Stabilizing tumor-resident mast cells restores T-cell infiltration and sensitizes sarcomas to PD-L1 inhibition. Clin Cancer Res. (2024) 30:2582–97. doi: 10.1158/1078-0432.CCR-24-0246, PMID: 38578281 PMC11145177

[126] Stylianopoulos T Jain RK. Combining two strategies to improve perfusion and drug delivery in solid tumors. Proc Natl Acad Sci U.S.A. (2013) 110:18632–7. doi: 10.1073/pnas.1318415110, PMID: 24167277 PMC3832007

[127] Chupradit K Muneekaew S Wattanapanitch M. Engineered CD147-CAR macrophages for enhanced phagocytosis of cancers. Cancer Immunol Immunother. (2024) 73:170. doi: 10.1007/s00262-024-03759-6, PMID: 38954079 PMC11219683

[128] Zhang W Liu L Su H Liu Q Shen J Dai H. Chimeric antigen receptor macrophage therapy for breast tumours mediated by targeting the tumour extracellular matrix. Br J Cancer. (2019) 121:837–45. doi: 10.1038/s41416-019-0578-3, PMID: 31570753 PMC6889154

[129] Chen Z Pan H Luo Y Yin T Zhang B Liao J. Nanoengineered CAR-T biohybrids for solid tumor immunotherapy with microenvironment photothermal-remodeling strategy. Small. (2021) 17:e2007494. doi: 10.1002/smll.202007494, PMID: 33711191

[130] Wang D Zhang M Qiu G Rong C Zhu X Qin G. Extracellular matrix viscosity reprogramming by *in situ* au bioreactor-boosted microwavegenetics disables tumor escape in CAR-T immunotherapy. ACS Nano. (2023) 17:5503–16. doi: 10.1021/acsnano.2c10845, PMID: 36917088

[131] Broz MT Ko EY Ishaya K Xiao J De Simone M Hoi XP. Metabolic targeting of cancer associated fibroblasts overcomes T-cell exclusion and chemoresistance in soft-tissue sarcomas. Nat Commun. (2024) 15:2498. doi: 10.1038/s41467-024-46504-4, PMID: 38509063 PMC10954767

[132] Li F Xu X Geng J Wan X Dai H. The autocrine CXCR4/CXCL12 axis contributes to lung fibrosis through modulation of lung fibroblast activity. xp Ther Med. (2020) 19:1844–54. doi: 10.3892/etm.2020.8433, PMID: 32104240 PMC7027131

[133] Chen IX Chauhan VP Posada J Ng MR Wu MW Adstamongkonkul P. Blocking CXCR4 alleviates desmoplasia, increases T-lymphocyte infiltration, and improves immunotherapy in metastatic breast cancer. Proc Natl Acad Sci U.S.A. (2019) 116:4558–66. doi: 10.1073/pnas.1815515116, PMID: 30700545 PMC6410779

[134] Canel M Taggart D Sims AH Lonergan DW Waizenegger IC Serrels A. T-cell co-stimulation in combination with targeting FAK drives enhanced anti-tumor immunity. Elife. (2020) 9:e48092. doi: 10.7554/eLife.48092, PMID: 31959281 PMC6974352

[135] Klabukov I Kabakov AE Yakimova A Baranovskii D Sosin D Atiakshin D. Tumor-associated extracellular matrix obstacles for CAR-T cell therapy: approaches to overcoming. Curr Oncol. (2025) 32(2):79. doi: 10.3390/curroncol32020079, PMID: 39996879 PMC11854105

[136] Liu T Liang H Li Y Liao W Deng J Zhang L. The increased matrix stiffness caused by LOXL2 activates Piezo1 channels to promote the migration and invasion of cervical cancer cells. Discov Oncol. (2025) 16:644. doi: 10.1007/s12672-025-02456-9, PMID: 40304808 PMC12044143

[137] Meng S Hara T Miura Y Ishii H. Fibroblast activation protein constitutes a novel target of chimeric antigen receptor T-cell therapy in solid tumors. Cancer Sci. (2024) 115:3532–42. doi: 10.1111/cas.16285, PMID: 39169645 PMC11531970

[138] Xin L Gao J Zheng Z Chen Y Lv S Zhao Z. Fibroblast activation protein-alpha as a target in the bench-to-bedside diagnosis and treatment of tumors: A narrative review. Front Oncol. (2021) 11:648187. doi: 10.3389/fonc.2021.648187, PMID: 34490078 PMC8416977

[139] Bughda R Dimou P D’Souza RR Klampatsa A. Fibroblast activation protein (FAP)-targeted CAR-T cells: launching an attack on tumor stroma. Immunotargets Ther. (2021) 10:313–23. doi: 10.2147/ITT.S291767, PMID: 34386436 PMC8354246

[140] Dickson PV Hamner JB Sims TL Fraga CH Ng CY Rajasekeran S. Bevacizumab-induced transient remodeling of the vasculature in neuroblastoma xenografts results in improved delivery and efficacy of systemically administered chemotherapy. Clin Cancer Res. (2007) 13:3942–50. doi: 10.1158/1078-0432.CCR-07-0278, PMID: 17606728

[141] Zhou Q Guo P Gallo JM. Impact of angiogenesis inhibition by sunitinib on tumor distribution of temozolomide. Clin Cancer Res. (2008) 14:1540–9. doi: 10.1158/1078-0432.CCR-07-4544, PMID: 18316579

[142] Zhao Q He X Qin X Liu Y Jiang H Wang J. Enhanced therapeutic efficacy of combining losartan and chemo-immunotherapy for triple negative breast cancer. Front Immunol. (2022) 13:938439. doi: 10.3389/fimmu.2022.938439, PMID: 35812418 PMC9259940

[143] Lanitis E Kosti P Ronet C Cribioli E Rota G Spill A. VEGFR-2 redirected CAR-T cells are functionally impaired by soluble VEGF-A competition for receptor binding. J Immunother Cancer. (2021) 9(8):e002151. doi: 10.1136/jitc-2020-002151, PMID: 34389616 PMC8365827

[144] Xie YJ Dougan M Jailkhani N Ingram J Fang T Kummer L. Nanobody-based CAR T cells that target the tumor microenvironment inhibit the growth of solid tumors in immunocompetent mice. Proc Natl Acad Sci U.S.A. (2019) 116:7624–31. doi: 10.1073/pnas.1817147116, PMID: 30936321 PMC6475367

[145] Carmona-Rodriguez L Martinez-Rey D Fernandez-Acenero MJ Gonzalez-Martin A Paz-Cabezas M Rodriguez-Rodriguez N. SOD3 induces a HIF-2alpha-dependent program in endothelial cells that provides a selective signal for tumor infiltration by T cells. J Immunother Cancer. (2020) 8(1):e000432. doi: 10.1136/jitc-2019-000432., PMID: 32591431 PMC7319787

[146] Carmona-Rodriguez L Martinez-Rey D Mira E Manes S. SOD3 boosts T cell infiltration by normalizing the tumor endothelium and inducing laminin-alpha4. Oncoimmunology. (2020) 9:1794163. doi: 10.1007/s00432-021-03895-x, PMID: 32934887 PMC7466848

[147] Zhang Z Yang N Xu L Lu H Chen Y Wang Z. Systemic delivery of oncolytic herpes virus using CAR-T cells enhances targeting of antitumor immuno-virotherapy. Cancer Immunol Immunother. (2024) 73:173. doi: 10.1007/s00262-024-03757-8, PMID: 38953982 PMC11219689

[148] Narayan V Barber-Rotenberg JS Jung IY Lacey SF Rech AJ Davis MM. PSMA-targeting TGFbeta-insensitive armored CAR T cells in metastatic castration-resistant prostate cancer: a phase 1 trial. Nat Med. (2022) 28:724–34. doi: 10.1038/s41591-022-01726-1, PMID: 35314843 PMC10308799

[149] Jan M Scarfo I Larson RC Walker A Schmidts A Guirguis AA. Reversible ON- and OFF-switch chimeric antigen receptors controlled by lenalidomide. Sci Transl Med. (2021) 13(575):eabb6295. doi: 10.1126/scitranslmed.abb6295, PMID: 33408186 PMC8045771

[150] Park HB Kim KH Kim JH Kim SI Oh YM Kang M. Improved safety of chimeric antigen receptor T cells indirectly targeting antigens via switchable adapters. Nat Commun. (2024) 15:9917. doi: 10.1038/s41467-024-53996-7, PMID: 39557825 PMC11574259

[151] Fang J Ding N Guo X Sun Y Zhang Z Xie B. alphaPD-1-mesoCAR-T cells partially inhibit the growth of advanced/refractory ovarian cancer in a patient along with daily apatinib. J Immunother Cancer. (2021) 9(2):e001162. doi: 10.1136/jitc-2020-001162, PMID: 33589520 PMC7887368

[152] Suarez ER Chang de K Sun J Sui J Freeman GJ Signoretti S. Chimeric antigen receptor T cells secreting anti-PD-L1 antibodies more effectively regress renal cell carcinoma in a humanized mouse model. Oncotarget. (2016) 7:34341–55. doi: 10.18632/oncotarget.9114, PMID: 27145284 PMC5085160

[153] Good CR Aznar MA Kuramitsu S Samareh P Agarwal S Donahue G. An NK-like CAR T cell transition in CAR T cell dysfunction. Cell. (2021) 184:6081–100:e6026. doi: 10.1016/j.cell.2021.11.016, PMID: 34861191 PMC8827167

[154] Sasaki T Sakoda Y Adachi K Tokunaga Y Tamada K. Therapeutic effects of anti-GM2 CAR-T cells expressing IL-7 and CCL19 for GM2-positive solid cancer in xenograft model. Cancer Med. (2023) 12:12569–80. doi: 10.1002/cam4.5907, PMID: 37031457 PMC10278466

[155] Stach M Ptackova P Mucha M Musil J Klener P Otahal P. Inducible secretion of IL-21 augments anti-tumor activity of piggyBac-manufactured chimeric antigen receptor T cells. Cytotherapy. (2020) 22:744–54. doi: 10.1016/j.jcyt.2020.08.005, PMID: 32950390

[156] Wang D Shao Y Zhang X Lu G Liu B. IL-23 and PSMA-targeted duo-CAR T cells in Prostate Cancer Eradication in a preclinical model. J Transl Med. (2020) 18:23. doi: 10.1186/s12967-019-02206-w, PMID: 31937346 PMC6961333

[157] Jones DS Nardozzi JD Sackton KL Ahmad G Christensen E Ringgaard L. Cell surface-tethered IL-12 repolarizes the tumor immune microenvironment to enhance the efficacy of adoptive T cell therapy. Sci Adv. (2022) 8:eabi8075. doi: 10.1126/sciadv.abi8075, PMID: 35476449 PMC9045725

[158] Wang X Zhao X Feng C Weinstein A Xia R Wen W. IL-36gamma transforms the tumor microenvironment and promotes type 1 lymphocyte-mediated antitumor immune responses. Cancer Cell. (2015) 28:296–306. doi: 10.1016/j.ccell.2015.07.014, PMID: 26321222 PMC4573903

[159] Zhu Y Wang K Yue L Zuo D Sheng J Lan S. Mesothelin CAR-T cells expressing tumor-targeted immunocytokine IL-12 yield durable efficacy and fewer side effects. Pharmacol Res. (2024) 203:107186. doi: 10.1016/j.phrs.2024.107186, PMID: 38641176

[160] Ohta K Sakoda Y Adachi K Shinozaki T Nakajima M Yasuda H. Therapeutic efficacy of IL7/CCL19-expressing CAR-T cells in intractable solid tumor models of glioblastoma and pancreatic cancer. Cancer Res Commun. (2024) 4:2514–24. doi: 10.1158/2767-9764.CRC-24-0226, PMID: 39240078 PMC11423281

[161] Zhu X Chen J Li W Xu Y Shan J Hong J. Hypoxia-responsive CAR-T cells exhibit reduced exhaustion and enhanced efficacy in solid tumors. Cancer Res. (2024) 84:84–100. doi: 10.1158/0008-5472.CAN-23-1038, PMID: 37874330

[162] Hatae R Kyewalabye K Yamamichi A Chen T Phyu S Chuntova P. Enhancing CAR-T cell metabolism to overcome hypoxic conditions in the brain tumor microenvironment. JCI Insight. (2024) 9(7):e177141. doi: 10.1172/jci.insight.177141, PMID: 38386420 PMC11128202

[163] Zhao Y Chen J Andreatta M Feng B Xie YQ Wenes M. IL-10-expressing CAR T cells resist dysfunction and mediate durable clearance of solid tumors and metastases. Nat Biotechnol. (2024) 42:1693–704. doi: 10.1038/s41587-023-02060-8, PMID: 38168996

[164] Lai J Mardiana S House IG Sek K Henderson MA Giuffrida L. Adoptive cellular therapy with T cells expressing the dendritic cell growth factor Flt3L drives epitope spreading and antitumor immunity. Nat Immunol. (2020) 21:914–26. doi: 10.1038/s41590-020-0676-7, PMID: 32424363

[165] Zhang Q Hresko ME Picton LK Su L Hollander MJ Nunez-Cruz S. A human orthogonal IL-2 and IL-2Rbeta system enhances CAR T cell expansion and antitumor activity in a murine model of leukemia. Sci Transl Med. (2021) 13:eabg6986. doi: 10.1126/scitranslmed.abg6986, PMID: 34936380 PMC9116279

[166] Aspuria PJ Vivona S Bauer M Semana M Ratti N McCauley S. An orthogonal IL-2 and IL-2Rbeta system drives persistence and activation of CAR T cells and clearance of bulky lymphoma. Sci Transl Med. (2021) 13:eabg7565. doi: 10.1126/scitranslmed.abg7565, PMID: 34936383

[167] Hyrenius-Wittsten A Su Y Park M Garcia JM Alavi J Perry N. SynNotch CAR circuits enhance solid tumor recognition and promote persistent antitumor activity in mouse models. Sci Transl Med. (2021) 13(591):eabd8836. doi: 10.1126/scitranslmed.abd8836, PMID: 33910981 PMC8594452

[168] Lee EHJ Murad JP Christian L Gibson J Yamaguchi Y Cullen C. Antigen-dependent IL-12 signaling in CAR T cells promotes regional to systemic disease targeting. Nat Commun. (2023) 14:4737. doi: 10.1038/s41467-023-40115-1, PMID: 37550294 PMC10406808

[169] Wang Y Fang X Li M Ye J Zhao S Yu L. Mesothelin CAR-T cells secreting PD-L1 blocking scFv for pancreatic cancer treatment. Cancer Genet. (2022) 268-269:103–10. doi: 10.1016/j.cancergen.2022.10.003, PMID: 36288641

[170] Agliardi G Liuzzi AR Hotblack A De Feo D Nunez N Stowe CL. Intratumoral IL-12 delivery empowers CAR-T cell immunotherapy in a pre-clinical model of glioblastoma. Nat Commun. (2021) 12:444. doi: 10.1038/s41467-020-20599-x, PMID: 33469002 PMC7815781

[171] Uslu U Sun L Castelli S Finck AV Assenmacher CA Young RM. The STING agonist IMSA101 enhances chimeric antigen receptor T cell function by inducing IL-18 secretion. Nat Commun. (2024) 15:3933. doi: 10.1038/s41467-024-47692-9, PMID: 38730243 PMC11087554

[172] Xu N Palmer DC Robeson AC Shou P Bommiasamy H Laurie SJ. STING agonist promotes CAR T cell trafficking and persistence in breast cancer. J Exp Med. (2021) 218(2):e20200844. doi: 10.1084/jem.20200844, PMID: 33382402 PMC7780733

